# Palladium-scavenging self-assembled hybrid hydrogels – reusable highly-active green catalysts for Suzuki–Miyaura cross-coupling reactions†
†Electronic supplementary information (ESI) available: Full experimental methods, UV-vis and rheology data, TEM imaging, characterization of all products and copies of ^1^H and ^13^C spectra. See DOI: 10.1039/c8sc04561e


**DOI:** 10.1039/c8sc04561e

**Published:** 2018-10-30

**Authors:** Petr Slavík, Dustin W. Kurka, David K. Smith

**Affiliations:** a Department of Chemistry , University of York , Heslington , York , YO10 5DD , UK . Email: david.smith@york.ac.uk

## Abstract

A hybrid hydrogel based on 1,3:2,4-dibenzylidene sorbitol (DBS) modified with acyl hydrazides combined with agarose was used for *in situ* reduction and binding of palladium from aqueous mixtures without the need for an external reducing agent. Palladium uptake was monitored and the formation of Pd nanoparticles (PdNPs) trapped within the gel and located close to the nanofibres was confirmed. This gel effectively scavenges palladium from solution to concentrations < 0.04 ppm – well below the recommended limits for pharmaceutical products. The resulting hybrid hydrogel with embedded PdNPs was used as a catalyst for Suzuki–Miyaura cross-coupling reactions. The gel network stabilises PdNPs, preventing aggregation/leaching and giving excellent catalytic lifetimes. The gel acts as a simple reaction dosing form, being simply added to reactions performed in green solvents in air. Once reactions are complete, the gel can be simply removed, recycled and reused (>10 times). Reactions were purified by simple washing protocols, and leaching of Pd from the gels is limited (<1 ppm). The gels were also used in flow-through mode, giving efficient, rapid reactions, with easy work-up. These catalytic gels combine advantages of homogeneous and heterogeneous catalysts – they are solvent compatible with the reaction taking place in a solution-like environment, while the solid-like gel network enables catalyst recycling. In summary, these hydrogels scavenge ‘waste’ palladium and convert it into gel ‘wealth’ capable of efficient, environmentally-friendly Suzuki–Miyaura catalysis.

## Abstract 2

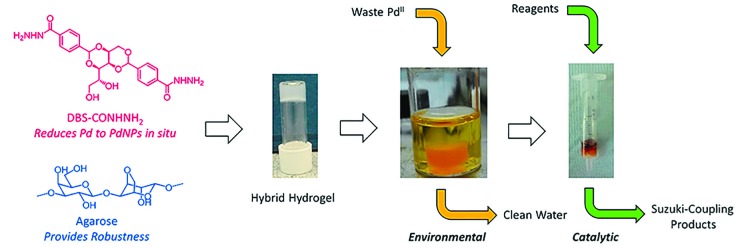
From waste to wealth – a self-assembled hydrogel remediates palladium from solution down to sub-ppm levels, and the resulting gel, which has embedded Pd nanoparticles, acts as a green and efficient catalyst for Suzuki–Miyaura cross-coupling reactions.

## Introduction

Supramolecular gels are colloidal soft materials based on the self-assembly of carefully-designed low-molecular-weight gelators (LMWGs) into a nanoscale solid-like network that spans a liquid-like phase.^1^ They have applications based on their rheological properties, and also have transformative potential in high-tech applications.^2^ Given that less than 1% of additive is typically able to immobilise bulk solvent, supramolecular gels are highly solvent compatible, porous materials, and small molecules can rapidly diffuse within them. Such gels therefore have significant potential in environmental remediation – polluted water can readily diffuse through hydrogels, with interactions between gel nanofibres and pollutants leading to effective pollutant immobilisation and hence removal.^3^ Furthermore, supramolecular gels have potential applications in catalysis.^4^ Reagents can diffuse into gels, products can diffuse out, and if the catalyst is immobilised within the network, it can be potentially removed and reused. As such, these materials are both homogeneous (solvent-compatible) and heterogeneous (solid-like), combining the advantages of both. Supramolecular gels can provide catalyst longevity, protection from trace product contamination and ease of catalyst separation/re-use.^4^ There is considerable potential to combine applications, and develop gels that can both remediate waste, and subsequently act as active catalysts – as yet, this has not been demonstrated.

Perhaps surprisingly, LMWGs have attracted relatively limited attention in precious metal-based catalysis. Most commonly, a palladium-binding ligand is incorporated into the gelator structure. Early work described gelators containing a pyridine moiety capable of binding Pd(OAc)_2_, with the resulting metallogels being used for the oxidation of benzylic alcohols.^5^ Later work used related systems to catalyse carbon–carbon bond forming reactions such as Suzuki–Miyaura cross coupling.^6^ James and co-workers prepared metal–organic gels using 5-diphenylphosphanylisophthalic acid as a palladium ligand, with the subsequent functionalized Pd-xerogel showing high catalytic activity in Suzuki cross-coupling.^7^ Another gel, based on palladium CNC pincer bis(carbene) complexes was reported by Dötz and co-workers and successfully used in Michael additions.^8^ Taking a different approach, Maitra and Maity used an external reducing agent to form palladium nanoparticles (PdNPs) in non-ligating calcium-cholate hydrogels,^9^ and showed that the dried xerogel could be used in Suzuki cross-coupling reactions. Unfortunately, the presence of K_2_CO_3_ partially destroyed the xerogel network. In terms of other catalytic metal NPs, Banerjee and co-workers have embedded AgNPs and AuNPs in gels to catalyse the reduction of nitroarenes to aminoarenes, but not PdNPs.^10^

Palladium has been a transformative catalytic metal, mediating a wide range of coupling reactions, yet is a finite resource, used in many applications.^11^ The recovery and reuse of this precious resource is of high value, in both economic and environmental terms. Scavenging Pd from waste is therefore of key importance.^12^ Furthermore, Pd is considered an unacceptable contaminant in pharmaceutical products – its use in synthesis must be carefully considered, and there is a need to sequester Pd contaminants.^13^ There has also been intense interest in developing ‘green’ syntheses using Pd to minimise environmental costs.^14^ With such issues in mind, we reasoned that supramolecular gels could scavenge Pd waste and the resulting gels could then act as heterogeneous supports in environmentally-friendly Pd-mediated coupling reactions – an integrated ‘waste-to-wealth’^15^ approach going significantly beyond other approaches to heterogeneous Pd catalysis.^16^

**Fig. 1:**
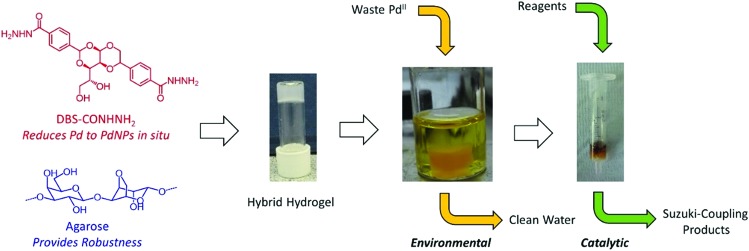
Schematic of the ‘waste-to-wealth’ approach using DBS-CONHNH_2_/agarose hybrid hydrogels to remediate waste, generating PdNPs *in situ* and then using the resulting material to catalyse Suzuki cross-coupling reactions.

Our novel hydrogelator, DBS-CONHNH_2_,^17^ based on the commercially-relevant, low-cost 1,3:2,4-dibenzylidene sorbitol (DBS) framework,^18^ has been shown to extract precious metals from model waste and immobilise them within the gel as a result of *in situ* reduction into precious metal NPs.^19^ In the case of gold extraction, the resulting gels exhibited unique conductance properties and could be further used for the modification of electrode surfaces and in electrocatalysis.^19^ We reasoned that in the case of palladium, the resulting materials may have the ability to be used in catalysis for organic synthesis. We therefore set out to fully understand the ability of DBS-CONHNH_2_ to scavenge palladium, and to use the resulting materials in organic synthesis – from waste to wealth (Fig. 1).

## Results and discussion

### Synthesis of DBS-CONHNH_2_ hybrid hydrogels with PdNPs

Hydrogelator DBS-CONHNH_2_ was prepared according to the previously published two-step procedure,17*a**via* acid-catalysed condensation of d-sorbitol with two equivalents of methyl-4-formylbenzoate and subsequent reaction of the methyl ester with hydrazine monohydrate. To improve the mechanical properties of these gels and facilitate handling, we formulated DBS-CONHNH_2_ with another component, agarose polymer gel (PG) – this hybrid PG/LMWG strategy, is an effective way of enhancing materials performance of rheologically weak supramolecular gels.^20^ These two components assemble independently of one another,17*d* with DBS-CONHNH_2_ providing the required functionality for precious metal capture and agarose providing mechanical robustness. We have previously performed rheological characterisation of this type of hybrid gel, with the studies indicating that the presence of agarose increases the *G*′ value by an order of magnitude.17*d*

**Fig. 2:**
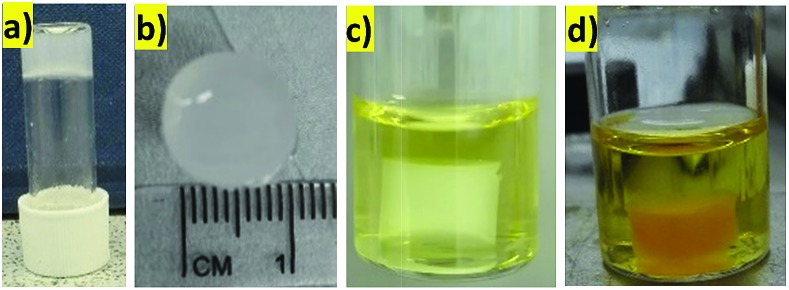
The preparation of Pd-hybrid hydrogels: (a) inversion test; (b) size of the gel; (c) immersion in PdCl_2_ solution; (d) hybrid gel with Pd loaded after 48 hours.

The hybrid hydrogel was simply formed using a simple heat-cool cycle (Fig. 2a and b) as both LMWG and PG are thermally responsive. Initially, to incorporate PdNPs inside the gel, blocks of the hybrid hydrogel (formed from 2.00 mg of DBS-CONHNH_2_, 2.50 mg of agarose and 0.55 mL of deionised water) were immersed in an aqueous solution of PdCl_2_ (5 mM) and allowed to stand for 48 hours at room temperature (Fig. 2c). The diffusion of Pd^2+^ into the gel and the formation of PdNPs within the gel were clearly visualised by a colour change from transparent to yellow/brown (Fig. 2d). Rheology indicated that for hybrid gels at this loading, the presence of PdNPs made the materials slightly stiffer (the *G*′ value roughly doubled, Fig. S5 and S6†). The gels were slightly more resistant to shear strain (*ca.* 2%) in the presence of PdNPs. Nanoparticles are known to enhance polymer rheological performance,^21^ but the effects seen here are only small. Most importantly, the gel-like nature of the materials in the presence of PdNPs was clearly maintained.

To quantify the amount of Pd inside the gels and gain insight into the rate of uptake, we studied extraction by UV-Vis spectroscopy (Fig. S1–S4, Tables S1–S3†). PdCl_2_ displays a strong absorption peak in the visible region at approx. *λ*_max_ = 425 nm that decreased over time during extraction. From the resulting concentration of Pd^2+^ in solution we could determine the amount of Pd loaded into the gel, which was typically 7–8 μmol. This implies that one equivalent of DBS-CONHNH_2_ is capable of reducing two equivalents of palladium, suggesting each acylhydrazide is responsible for the reduction of one equivalent of palladium. Two further experiments supported the existence of a 1 : 1 relationship between Pd and the acyl hydrazide. Doubling the concentration of PdCl_2_ from 5 mM to 10 mM led to only very small increases of Pd within the gel (aprox. 10.5 μmol), suggesting that the system was effectively saturated. On the other hand, halving the loading of DBS-CONHNH_2_ hydrogelator (from 2.00 mg to 1.00 mg) resulted in *ca.* half the amount of Pd inside the gel (5 μmol). To determine the influence of the temperature on Pd uptake, the extraction experiment was also performed at 50 °C. The total loading of Pd within the gel remained the same, but uptake was *ca.* two times faster (see ESI†). At room temperature, after one hour, the loading was 2.2 μmol, whereas at 50 °C it was 3.9 μmol.

It is worth noting here that agarose alone can also be used as a gel support/ligand for PdNPs, but external reducing agents such as citric acid are necessary for NP formation.^22^ Furthermore, the literature reports indicate that a maximum palladium loading of just 0.1 mmol g^–1^ onto agarose can be achieved. This compares with the 1.8 mmol g^–1^ onto agarose/DBS-CONHNH_2_ achieved by us here. This 18-fold enhancement clearly demonstrates the active role played by DBS-CONHNH_2_ in enabling efficient loading of precious metal NPs into the hybrid hydrogel.

We then tested the ability of these gels to scavenge Pd from solution at lower concentrations – as this demonstrates their potential use in the clean-up of waste streams. The resulting solution after uptake was analysed by Atomic Absorption Spectroscopy (AAS). For example, when a 0.83 mM PdCl_2_ solution (3.6 mL) was brought into contact with the gel block, the resulting concentration of Pd in solution after 48 hours was below the AAS detection limit (<0.04 ppm). This suggests that >99.97% of Pd became embedded within the gel, indicating an outstanding ability of this hybrid hydrogel to scavenge Pd^II^ from waste.

**Fig. 3:**
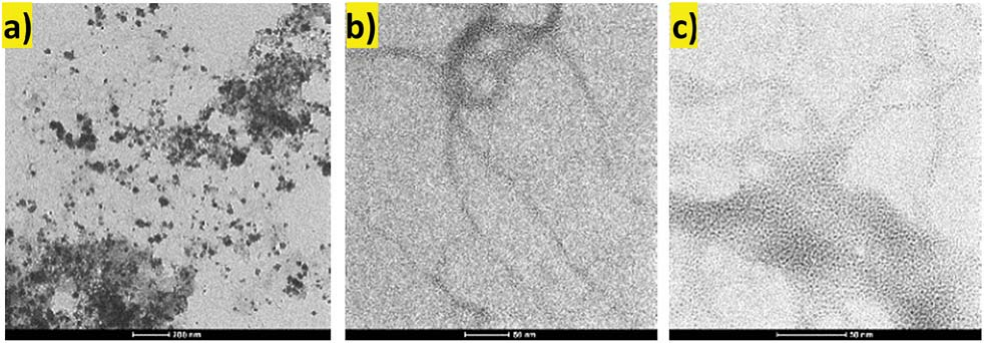
TEM images of PdNPs hybrid hydrogel. Scale bars: (a) 200 nm; (b) and (c) 50 nm.

Transmission electron microscopy (TEM) indicated that the PdNPs formed during loading of the gel are not randomly distributed within the gel, but appear to be in close proximity to the gel fibres (Fig. 3). This is in agreement with a mechanism in which the acyl hydrazides are oxidised^19^,^23^ and hence mediate reduction of Pd^2+^ to Pd^0^, with the resulting nanoparticles having limited mobility within the gel network. We suggest that electrostatic interactions between the hydrazide functionalised gel fibres and electron-deficient Pd^2+^ ions lead to reduction on the periphery of the gel nanofibers. TEM indicates that the PdNPs are mostly spherical and the average size is <5 nm. We propose that the PdNPs generated in this way will be effectively “naked” un-capped particles (no explicit stabilising ligand is added), which should make them highly catalytically active (see below). The network of the gel should also prevent NP aggregation, limit problems such as catalyst leaching and hence improve catalyst recyclability (see below).

### Cross-coupling reactions using PdNPs/DBS-CONHNH_2_ as a catalyst

We then went on to demonstrate that these Pd-loaded gels were catalytically proficient in a key synthetic methodology – Suzuki–Miyaura cross-coupling. In this way, high-value-added applications can be achieved using materials that have been scavenging for palladium waste. Cross-coupling reactions are most usually performed in organic solvents under an inert atmosphere with various ligands that can increase catalyst activity,^11^ although there has been significant work on developing more environmentally-friendly approaches^14^ and some ligand-free cross-coupling reactions are also known.^24^ Due to the fact that in our hybrid hydrogels the PdNPs are stabilised by the gel network, we carried out cross-coupling reactions without any additional ligands.

Optimal Suzuki–Miyaura reaction conditions were established by monitoring the coupling reaction of 4-iodotoluene with phenylboronic acid (Table 1). Reactions were performed in a vial without stirring to avoid mechanical degradation of the hybrid hydrogel. Due to the increasing importance of green solvents in organic synthesis, we tried to avoid traditionally-used organic solvents, such as toluene or THF. Best results were obtained for reactions performed in a mixture of ethanol and water (3 : 1) that enables both efficient cross-coupling reaction and easy separation of the products. At room temperature, 95% yield was obtained after 144 hours (entry 1). At 50 °C, a similar yield (95%) was obtained after 18 hours (entry 2). Further increasing the reaction temperature to 70 °C did not result in any significant enhancement of reaction rate (entry 3). Higher temperatures were not used because of limitations of gel stability. As can be seen from entries 2, 5 and 6, the choice of base did not have any significant influence on the progress of the reaction. On the other hand, if reactions were performed without any base, very low yields (16%) were obtained (entry 4). Therefore, K_2_CO_3_ was selected as the most suitable base as it is low-cost and environmentally benign. We attempted to reduce the reaction time further, but this was not easily possible. We suggest diffusion in and out of the gel limits the rate. To test the influence of the agarose on the hybrid-hydrogel, we performed the same cross-coupling reaction with just the DBS-CONHNH_2_ hydrogel loaded with Pd. This gave similar results as with the hybrid-hydrogel, but the gels were not so easily handled. This suggests that role of the agarose is mostly mechanical, but small contributions to catalytic function cannot be fully ruled out.^22^

**Table 1:** Initial screening of the reaction conditions for the Suzuki coupling of 4-iodotoluene and phenylboronic acid^*a*^

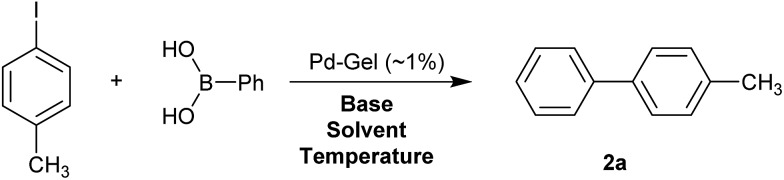					
Entry	Base	Solvent	*T* [°C]	Time	Yield^*b*^ [%]
1	K_2_CO_3_	EtOH/H_2_O (3 : 1)	r.t.	144 h	95
2	K_2_CO_3_	EtOH/H_2_O (3 : 1)	50	18 h	94
3	K_2_CO_3_	EtOH/H_2_O (3 : 1)	70	18 h	94
4	None	EtOH/H_2_O (3 : 1)	50	18 h	16
5	KOH	EtOH/H_2_O (3 : 1)	50	18 h	96^*c*^
6	Cs_2_CO_3_	EtOH/H_2_O (3 : 1)	50	18 h	95
7	K_2_CO_3_	H_2_O	50	18 h	Traces
8	K_2_CO_3_	EtOH	50	18 h	94

^*a*^Reaction conditions: 4-iodotoluene (0.46 mmol), phenylboronic acid (0.58 mmol), base (0.97 mmol), solvent (4 mL) and Pd-gel (∼1% mol of Pd). The reaction was carried out without stirring at a specified temperature.

^*b*^Isolated yield.

^*c*^Gel was partially destroyed during the reaction.

As a next step, we investigated the influence of Pd-loading on reaction progress (Table 2). As a control, the reaction using hybrid-hydrogel without any Pd did not yield any of the desired product (entry 1). On the other hand, using 1.5 mol% Pd gave 95% yield in 18 hours (entry 2). Similar yields were obtained for reactions with lower catalyst loadings (entries 3–5). However, for very low concentrations of Pd (0.01 mol%), longer reaction times were needed and the obtained yield was only 47% (entry 6). In further studies, 1 mol% Pd in gels was used to ensure equal distribution of the PdNPs and consistent reaction conditions.

**Table 2:** The effect of Pd loading on the Suzuki coupling of 4-iodotoluene and phenylboronic acid^*a*^

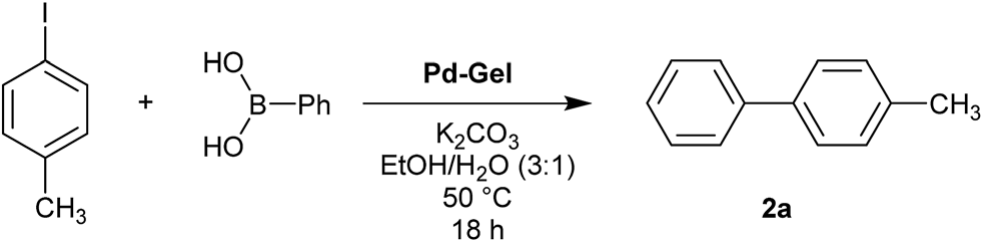		
Entry	Amount of Pd [mol%]	Yield^*b*^ [%]
1	0	None
2	1.5	95
3	1.0	94
4	0.1	95
5	0.05	93
6	0.01	47^*c*^

^*a*^Reaction conditions: 4-iodotoluene (0.46 mmol), phenylboronic acid (0.58 mmol), base (0.97 mmol), solvent (4 mL) and Pd-gel (∼1% mol of Pd). The reaction was carried out without stirring at a specified temperature.

^*b*^Isolated yield.

^*c*^After 43 h.

Reaction conditions were then further applied to the reactions of a broader range of functionalized aryl halides with phenylboronic acid (Table 3) to explore the scope of this methodology. In general, both electron rich (entries 1–3) and electron poor (entries 4–9) aryl iodides gave excellent yields (>90%). This included an aromatic amine – such functional groups are important in pharmaceutical applications. Moreover, in most cases, there was no need for any further purification except for removal of excess boronic acid, which was simply achieved by extracting the desired product into diethyl ether and washing with 1 M aq. NaOH and water. Importantly, the amount of residual palladium found in the crude product was below the AAS detection limit (<0.04 ppm), which easily meets the criteria for the oral concentration limit for medical products (<10 ppm) even without any further purification. This lack of catalyst contamination in the product demonstrates a clear advantage provided by the heterogeneous nature of the gel-based approach exemplified here.

**Table 3:** Suzuki coupling of aryl iodides with phenylboronic acid^*a*^

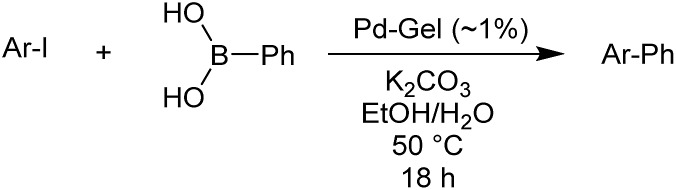			
Entry	Aryl iodide	Product	Yield^*b*^ [%]
1	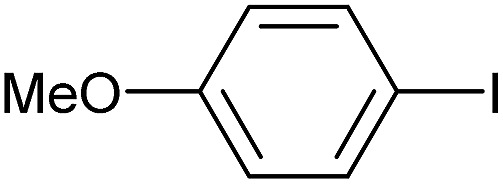	**2b**	94
2	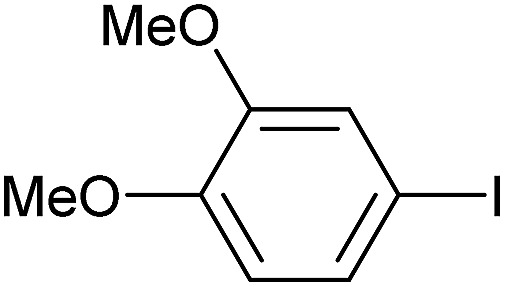	**2c**	100
3	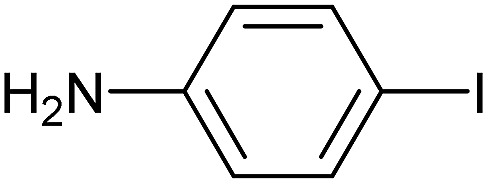	**2d**	93
4	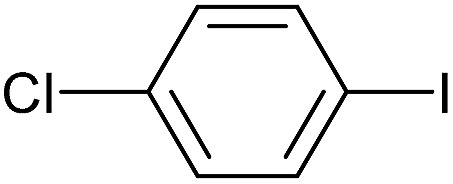	**2e**	91
5	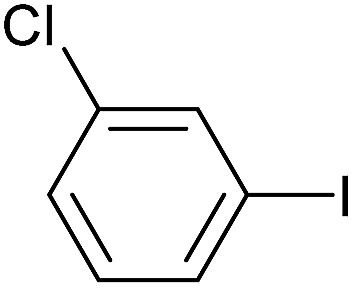	**2f**	91
6	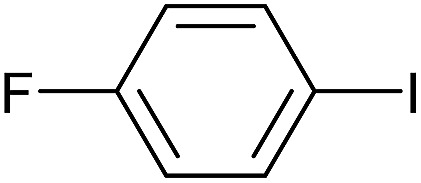	**2g**	97
7	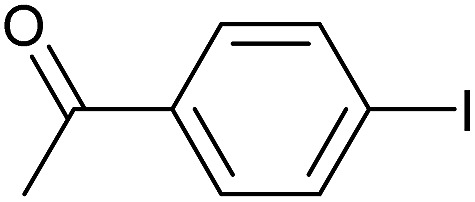	**2h**	86
8	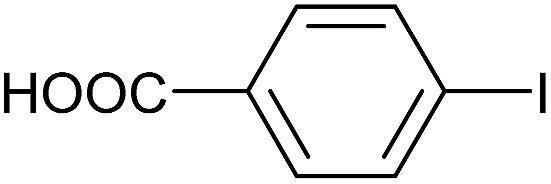	**2i**	98^*c*^
9	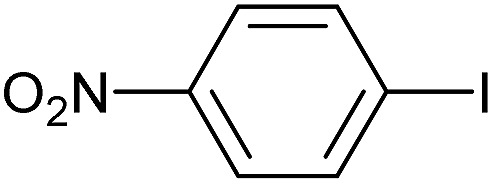	**2j**	97
10	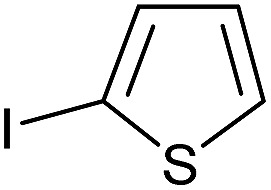	**2k**	92
11	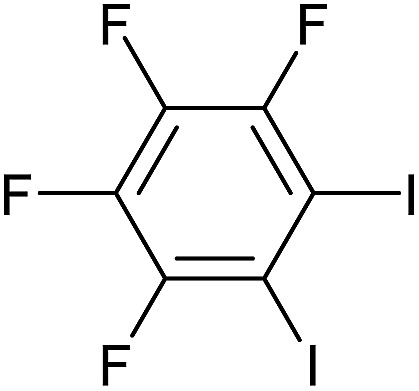	**2l**	69^*d*^

^*a*^Reaction conditions: aryl iodide (0.80 mmol), phenylboronic acid (0.96 mmol), K_2_CO_3_ (1.60 mmol), EtOH (3 mL), H_2_O (1 mL) and Pd-gel (∼1 mol% of Pd). The reaction was carried out without stirring at 50 °C. Reaction times were not minimized.

^*b*^Isolated yield.

^*c*^0.81 mmol of phenylboronic acid was used.

^*d*^Reaction conditions: aryl iodide (0.80 mmol), phenylboronic acid (1.92 mmol), K_2_CO_3_ (3.84 mmol), EtOH (3 mL), H_2_O (1 mL) and Pd-gel (∼1 mol% of Pd). The reaction was carried out without stirring at 50 °C.

To further investigate the scope of this reaction, we tried to couple various aryl bromides and chlorides (Table 4). In the case of aryl bromides, the reactivity remained almost the same as with aryl iodides (entries 1–3). However, the reaction of aryl chlorides provided poor yields, even with prolonged reaction times (entries 4 and 5). These results are in agreement with literature data for cross-coupling reactions,14*c* and in accord with the relative strengths of the C–X bond, which must be broken in the rate-determining step of the catalytic cycle.^25^ We also explored several different boronic acids (data not tabulated here) and determined that couplings using an alternative aromatic boronic acid (*p*-methoxyphenyl boronic acid) worked well, whereas the use of an aliphatic boronic acid (butylboronic acid) was unsuccessful.

**Table 4:** Suzuki coupling of aryl bromides and chlorides with phenylboronic acid^*a*^

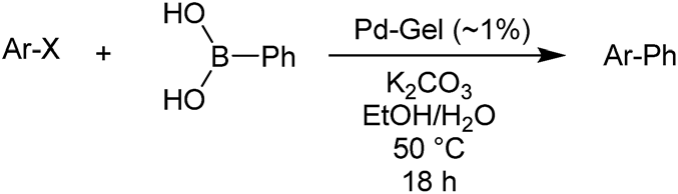			
Entry	Aryl halide	Product	Yield^*b*^ [%]
1	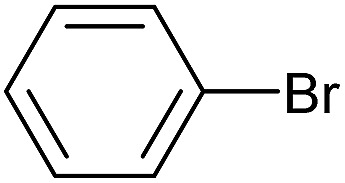	**2m**	86
2	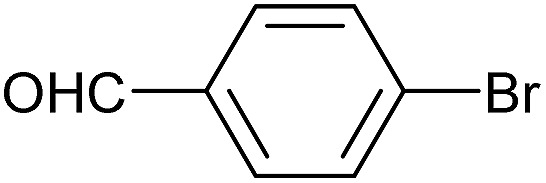	**2n**	95^*c*^
3	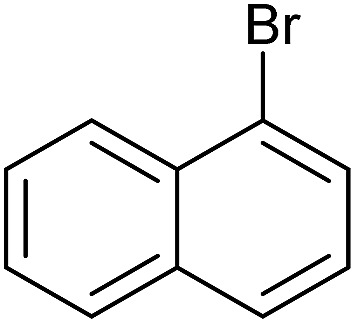	**2o**	96
4	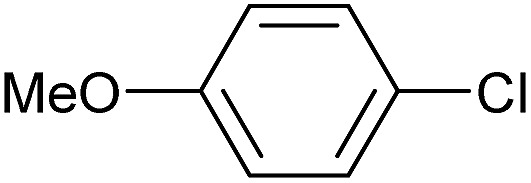	**2b**	24^*d*^
5	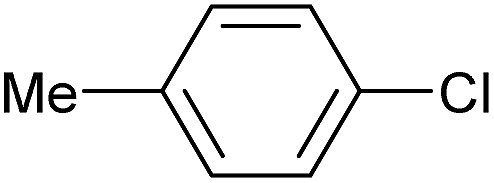	**2a**	Traces^*d*^

^*a*^Reaction conditions: aryl halide (0.80 mmol), phenylboronic acid (0.96 mmol), K_2_CO_3_ (1.60 mmol), EtOH (3 mL), H_2_O (1 mL) and Pd-gel (∼1 mol% of Pd). The reaction was carried out without stirring at 50 °C. Reaction times were not minimized.

^*b*^Isolated yield.

^*c*^0.81 mmol of phenylboronic acid used.

^*d*^After 72 h.

We also scaled up our standard reaction (4-iodotoluene with phenylboronic acid) to 5 mmol, in order to test the feasibility of this process at larger scale. For this purpose, a larger hybrid hydrogel was prepared (12.50 mg of DBS-CONHNH_2_, 15.63 mg of agarose, 3.44 mL of H_2_O, 1% mol of Pd). The desired product **2a** was isolated in nearly identical yield (91%) to those previously described, although, a longer reaction time (24 hours) was necessary to reach full conversion. Again, we suggest diffusion of reagents into the gel limits rate – one way to circumvent this would be to dose larger-scale reactions with multiple smaller gel blocks.

### Catalyst recyclability

Since one of the main advantages of our hybrid hydrogels with PdNPs should be ease of recyclability, we performed a series of reactions between 4-iodotoluene and phenylboronic acid to investigate this. After completion of each run, the product was extracted with diethyl ether and the hybrid hydrogel was simply removed from the reaction vial with a spatula, washed with diethyl ether to ensure extraction of all the product, and then washed with deionized water so it is compatible with the aqueous solvent environment and is ready to be used directly in the subsequent reaction. As such, this gel constitutes a very easily recycled reaction-dosing form – even formal filtration is not required.

As shown in Table 5, no significant change of catalyst activity was observed during the first eleven consecutive runs. After the 11^th^ run, there was a small loss of activity (run 12–14) most probably caused by mechanical degradation of the catalytic gel. In total, only *ca.* 0.8 mg of Pd produced more than 1.7 g of the desired product **2a** across this series of repeated reactions.

**Table 5:** Suzuki reaction of 4-iodotoluene and phenylboronic acid using recycled Pd-gel^*a*^

Entry	Yield^*b*^ [%]	Conversion^*c*^ [%]
1	97	100
2	98	100
3	99	100
4	99	100
5	99	100
6	99	100
7	98	100
8	95	100
9	97	100
10	98	100
11	96	100
12	86	95
13	78	93^*d*^
14	74	86^*e*^

^*a*^Reaction conditions: 4-iodotoluene (0.80 mmol), phenylboronic acid (0.96 mmol), K_2_CO_3_ (1.60 mmol), EtOH (3 mL), H_2_O (1 mL) and Pd-gel (∼1 mol% of Pd). The reaction was carried out without stirring at 50 °C for 18 h. Reaction times were not minimized.

^*b*^Isolated yield.

^*c*^Determined by ^1^H NMR.

^*d*^After 25 h.

^*e*^After 26.5 h.

**Fig. 4:**
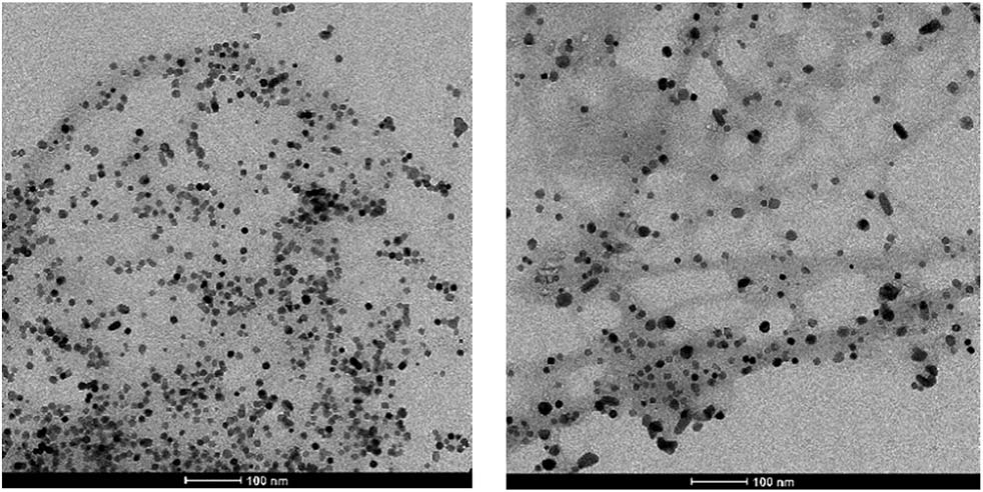
TEM image of the recycled catalyst after first (left) and fifth (right) run at 50 °C. Scale bars 100 nm.

TEM imaging of the recycled and reused catalyst after five runs was similar to that of the fresh catalyst (Fig. 4). However, some limited aggregation of palladium nanoparticles was observed, probably due to the influence of elevated reaction temperature – average PdNP size was *ca.* 10 nm (Fig. S7†). Indeed, raising the temperature of these gels was observed to cause a small amount of PdNP aggregation even in the absence of reaction (Fig. S8†). The recyclability study and TEM images of used catalyst suggest that the amount of Pd leaching into the reaction mixture must be negligible. This was further proven by AAS analysis of the hot reaction mixture (conversion *ca.* 50%) which quantified the concentration of Pd in solution as only 0.7 ppm – importantly, this is well below the required level for medical products.

To further prove the heterogeneous nature of the catalyst, we performed a hot filtration test.^26^ The reaction of 4-iodotoluene, phenylboronic acid, K_2_CO_3_ and Pd-gel catalyst (1% mol) was left to react for 24 hours and then the hot reaction mixture was filtered using a nylon syringe filter (0.22 μm). Another reaction between 4-iodoanisole (0.60 mmol) and phenylboronic acid (0.72 mmol) was directly carried out in the filtrate. After 24 hours, the crude reaction mixture was analysed by ^1^H NMR and a small amount (34%) of conversion was observed. This indicates that the small amount of palladium in the solution (in good general agreement with AAS studies) means the reaction can be partly homogeneously catalysed. However, since conversion is much less than when the hybrid hydrogel catalyst is present (34% *vs.* complete conversion), we conclude catalysis is mostly heterogeneous.

### Reaction in a flow-through device

**Fig. 5:**
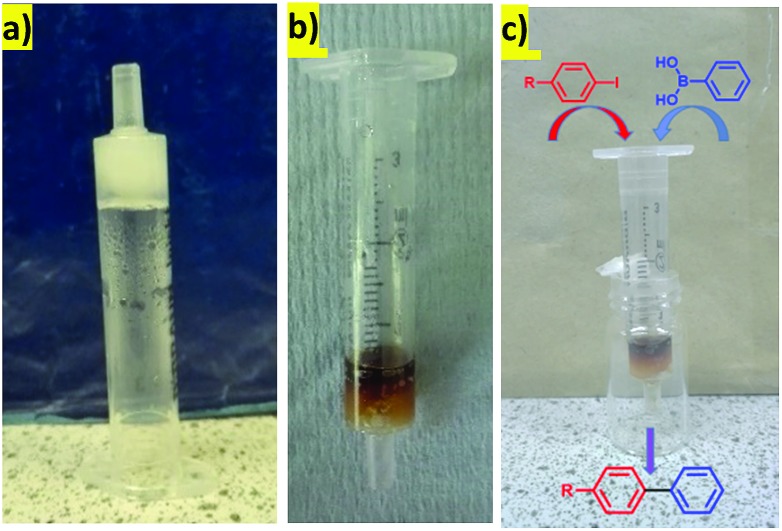
The hybrid-hydrogel in a syringe: (a) before Pd uptake; (b) after Pd uptake; (c) schematic representation of the reaction.

Heterogeneous catalysts are often used in flow-through devices that offer many advantages over commonly used reaction setups, such as enhanced heat and mass transfer, possibilities of scale-up, or specified control over reaction and retention times.^27^ We were therefore interested to determine whether our hydrogels with PdNPs could be used in this way. We prepared a very simple flow-through device made from a plastic syringe. In a typical experiment, a 3 mL plastic syringe was partly blocked with cotton-wool at the bottom and a hot hydrosol (made from 2.0 mg of DBS-CONHNH_2_ and 0.65 mL of deionised water) was transferred on the top. The hydrosol was allowed to cool down at room temperature as the hybrid hydrogel formed (Fig. 5a). PdNPs were embedded within the gel in a similar way as described before (by the interaction with a solution of PdCl_2_, Fig. 5b). This very simple device was then directly used for the Suzuki coupling experiments in the flow-through mode illustrated (Fig. 5c) under the influence of gravity.

We initially studied our standard cross-coupling reaction between 4-iodotoluene and phenylboronic acid (Table 6, entry 1). The reaction conditions were almost identical to those used in the normal reaction setup. The only difference was the use of KOH as base instead of K_2_CO_3_ (due to the shorter reaction times in flow-through mode). The reaction was performed in an incubator at 50 °C to ensure that the temperature was the same in all parts of the gel. When the reaction was performed with a very fast flow-rate *ca.* 9 mL min^–1^ (complete diffusion through gel was finished in 20 seconds), conversion was 70%. Since these gels are relatively fragile (no agarose PG is present in these experiments), we were not able to apply any pressure and thus, the flow-rates varied significantly, and could not be directly controlled.

**Table 6:** Suzuki coupling in the flow-through syringe device

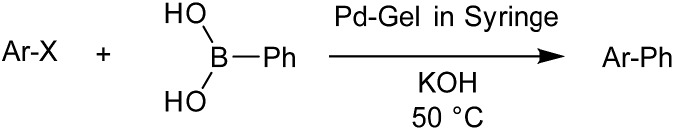				
Entry	Reagent	Flow rate [mL min^–1^]	Product	Conversion^*c*^ [Yield^*d*^, %]
1^*a*^	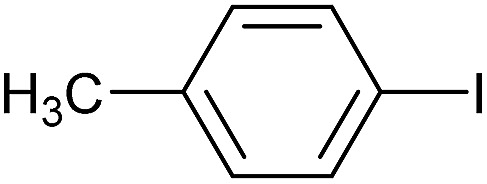	9	**2a**	70
2^*a*^	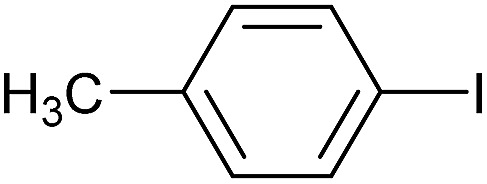	0.2	**2a**	100 [87]
3^*a*^	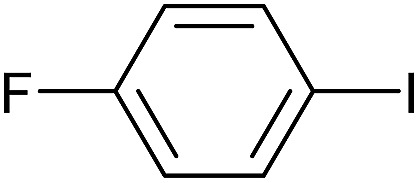	0.05	**2i**	100 [95]
4^*a*^	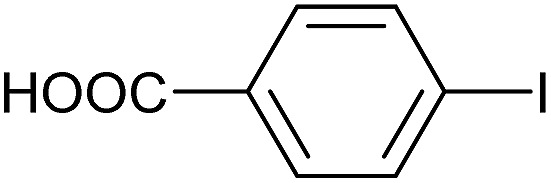	0.1	**2g**	100 [90]
5^*b*^	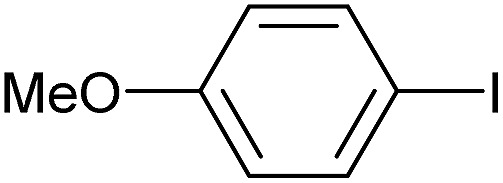	0.08	**2b**	100 [87]
6^*b*^	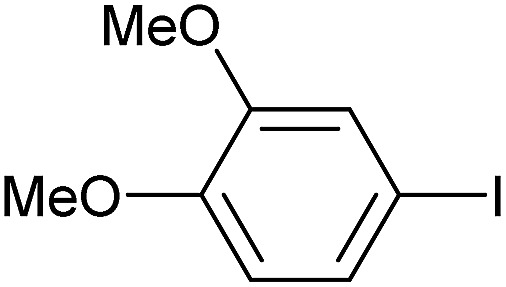	0.02	**2c**	100 [89]
7^*b*^	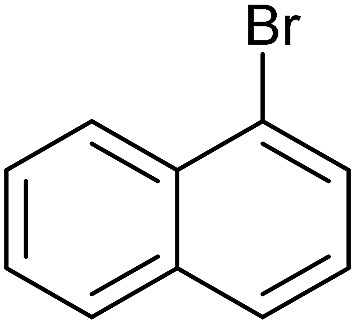	0.02	**2m**	98 [88]

^*a*^Reaction conditions: aryl iodide (0.40 mmol), phenylboronic acid (0.41 mmol), KOH (0.80 mmol), EtOH (3 mL), H_2_O (1 mL) and Pd-gel (∼1.5 mol% of Pd). The reaction was performed in the incubator at 50 °C.

^*b*^Reaction conditions: aryl iodide/bromide (0.80 mmol), phenylboronic acid (0.81 mmol), KOH (1.60 mmol), PEG 200 (1 mL) Pd-gel (∼1 mol% of Pd). The reaction was performed in the incubator at 50 °C.

^*c*^Determined by ^1^H NMR.

^*d*^Isolated yield in parentheses.

This fast flow rate was not typical – more usually flow rates 0.02–0.2 mL min^–1^ were observed (entries 2–7), with flow-through therefore being complete in 15–150 min. In these cases, near quantitative conversion was obtained, suggesting that with the faster flow rate described above, the gel may have been somewhat cracked, with not all of the reagent coming into contact with the immobilised catalyst. Clearly, however, our gels are highly active and can be efficiently used in a flow-through mode. As can be seen from Table 6, when these flow-through conditions were applied to a variety of substrates, in all experiments we obtained excellent yields in much shorter reaction times compared with the classical setup. Due to solubility issues with some products (entries 5–7), we changed solvent from an EtOH/H_2_O mixture to PEG 200. This resulted in a small drop in observed flow-rate, but the isolated yields were still excellent. In future work it may also be worth exploring other green solvents in this flow-through approach.

It is noteworthy that in flow-through mode the reaction is much faster than in the standard reaction set-up described earlier (Table 1). We suggest this is the result of the flow through the device under the force of gravity ensuring contact of reagents with the catalyst, and preventing the passive diffusion of reagents in and out of the gel from becoming rate limiting.

We then tested the agarose/DBS-CONHNH_2_ hybrid gel loaded with PdNPs in flow-through mode. This gave broadly similar results to DBS-CONHNH_2_ alone, however the flow rate was somewhat lower. We hoped that these gels may be strong enough to handle flow-through reactions under pressure, but unfortunately they were too fragile. Further work here will focus on optimising the PG component to combine with DBS-CONHNH_2_ such that these materials are even more robust,17*c* and can potentially be incorporated into columns. This should enable automation and more rapid flow-through reaction processes (*ca.* 1 min) that will become competitive with other literature reports.^28^ Our system also offers the significant advantage of working at relatively low temperatures.

Finally, having developed a simple flow-through device capable of quick filtration we wondered if this could also be used for Pd scavenging. A hydrogel (made from 3.1 mg of DBS-CONHNH_2_ and 0.75 mL of deionised water) was prepared in a 1 mL syringe and placed in the incubator at 50 °C. A solution of PdCl_2_ (0.7 mL, 2.86 mM) was added portion-wise to the top of the gel and allowed to flow through it. After complete filtration (*ca.* 20 min), the gel was further washed with 0.8 mL of deionised water. After this experiment, the colour of the gel had changed from transparent to orange indicating embedding of PdNPs (Fig. S9†). The collected filtrate was colourless, and was mixed with 3 mL of EtOH and then directly used as a solvent for the Suzuki coupling between 4-iodotoluene and phenylboronic acid. However, after 24 hours we did not detect any product. This implies that effectively all of the Pd from the initial solution (500 ppm) was successfully scavenged in real time (20 min) using this very simple flow-through device. We suggest this approach has real potential for cleaning up Pd from waste streams or pharmaceutical products.

## Conclusions

In conclusion, we have shown that DBS-CONHNH_2_ is capable of converting waste-to-wealth by scavenging palladium from solution, converting it *in situ* and without external reducing agent into nanoparticle form, and that the resulting gels then have the capacity to catalyse Suzuki–Miyaura cross-coupling reactions. Scavenging was able to remove palladium from solution down to <0.04 ppm levels – importantly far below the acceptable levels in pharmaceutical/medical products, suggesting potential uses for these gels in the clean-up of pharmaceutical processes and products. Suzuki–Miyaura reactions were also performed using green solvents (EtOH/H_2_O) with benign bases (K_2_CO_3_) and avoided the need for inert atmosphere conditions. Excellent yields together with high stability and easy recyclability make these gels efficient catalysts for this type of reaction. Limited Pd leaching was observed, with concentrations below the tolerance for pharmaceuticals, suggesting potential applications of these gels in high-value pharmaceutical synthesis. Furthermore, the use of agarose to provide the hybrid gels with physical robustness means that these catalytically-active gels can easily be dosed into reactions, recovered at the end by ‘fishing out’, and reused in further reactions. Moreover, DBS-CONHNH_2_ gels could be simply used in flow-through mode, giving rapid full conversion of reagents into products with easy purification. In summary, this research demonstrates that DBS-CONHNH_2_ is an effective way of scavenging ‘waste’ palladium and converting it into catalytic gel-phase ‘wealth’ capable of efficient, environmentally-friendly Suzuki–Miyaura reactions.

## Conflicts of interest

There are no conflicts to declare.

## Supplementary Material

Supplementary information
