# Correction: Palladium-scavenging self-assembled hybrid hydrogels – reusable highly-active green catalysts for Suzuki–Miyaura cross-coupling reactions

**DOI:** 10.1039/c8sc90226g

**Published:** 2018-11-09

**Authors:** Petr Slavík, Dustin W. Kurka, David K. Smith

**Affiliations:** a Department of Chemistry , University of York , Heslington , York , YO10 5DD , UK . Email: david.smith@york.ac.uk

## Abstract

Correction for ‘Palladium-scavenging self-assembled hybrid hydrogels – reusable highly-active green catalysts for Suzuki–Miyaura cross-coupling reactions’ by Petr Slavík *et al.*, *Chem. Sci.*, 2019, DOI: ; 10.1039/c8sc04561e.



## 

In the original article, an error was made in the placement of an oxygen atom in the structure of DBS-CONHNH_2_ in Fig. 1. The structure should be as shown below:

**Fig. 1:**
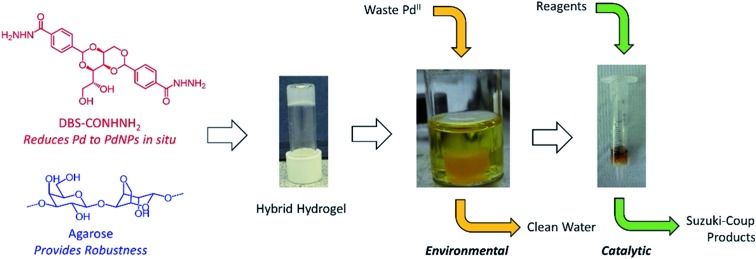
Schematic of the ‘waste-to-wealth’ approach using DBS-CONHNH_2_/agarose hybrid hydrogels to remediate waste, generating PdNPs *in situ* and then using the resulting material to catalyse Suzuki cross-coupling reactions.

The Royal Society of Chemistry apologises for these errors and any consequent inconvenience to authors and readers.

