# Supplementary material for: Palladium-scavenging self-assembled hybrid hydrogels – reusable highly-active green catalysts for Suzuki–Miyaura cross-coupling reactions
Source: Chem Sci. 2018 Oct 30;9(46):8673–81. doi: 10.1039/c8sc04561e (PMC6301269; doi:10.1039/c8sc04561e)
Supplement: Supplementary file 1 [file SC-009-C8SC04561E-s001.pdf]

Electronic Supplementary Material (ESI) for Chemical Science.
This journal is © The Royal Society of Chemistry 2018

Supporting Information for

Palladium-scavenging self-assembled hybrid hydrogels – reusable highly-active green catalysts for Suzuki-Miyaura cross-coupling reactions

Petr Slavík,[a] Dustin W. Kurka,[a] David K. Smith[a]

[a]Department of Chemistry, University of York, Heslington, York, YO10 5DD, United Kingdom

E-mail: david.smith@york.ac.uk

Table of Contents

1. Experimental procedures and characterizations p. S2

2. Preparation of Gels p. S2

3. Uptake of Pd^{2+} onto DBS-CONHNH$_2$ hybrid hydrogel p. S3-S4

4. Rheological Studies p. S5

5. General procedure for Suzuki cross-coupling reaction p. S6

6. Catalyst recyclability studies p. S6

7. Hot filtration test p. S6

8. Suzuki cross-coupling in a syringe p. S6-S7

9. Pd scavenging experiment in a flow-through device p. S7

10. Characterization data of the products p. S8-S10

11. Copies of ^{1}H and ^{13}C NMR spectra p. S11-25

12. References p. S26

1. Materials and methods

General Experimental Methods. All compounds required in synthesis and analysis were purchased from standard commercial suppliers. The synthesis of DBS-CONHNH$_2$ was performed using previously published procedure.[1] ^{1}H and ^{13}C NMR were recorded on a JEOL ECX 400 (^{1}H 400 MHz, ^{13}C 101 MHz) Spectrometer. Samples were recorded as solutions in deuterated NMR solvents as stated and chemical shifts (*δ*) are quoted in parts per million. UV-Vis spectroscopy was performed on a Shimadzu UV-2401 spectrometer. UV-Vis absorbance was measured on a Shimadzu UV-2401 PC spectrophotometer.

2. Preparation of Gels

Preparation of DBS-CONHNH$_2$ hydrogels. A known quantity of DBS-CONHNH$_2$ (usually 2.0 mg, 4.47 µmol) and agarose (usually 2.5 mg) were weighed into a 2.5 mL sample vial and 0.55 mL deionised water was added. The vial was then sonicated to disperse the solid. The mixture was heated with shaking until the gelator completely dissolved. The hydrosol was transferred into a removable-base vial (2 mL) with the base temporarily held on with parafilm (removable-base vial could be replaced with 5 mL plastic syringe that is partially cut). The hydrosol was allowed to cool down at room temperature as the hybrid hydrogel formed.

3. Uptake of Pd^{2+} onto DBS-CONHNH$_2$ hybrid hydrogel

Uptake of Pd^{2+}onto DBS-CONHNH$_2$ hybrid hydrogel was monitored by UV-VIS spectroscopy and the residual concentration was calculated from the calibration curve plotted for UV-VIS absorption of $PdCl_2$ at λ_{max}= 425 nm. All hybrid hydrogels were formed using 2.0 mg of DBS-CONHNH$_2$, 2.5 mg of agarose and 0.55 mL of deionised water. In order to study absorption of $PdCl_2$, hybrid hydrogels were transformed into a vial and left to interact with the $PdCl_2$ solution for desired time.

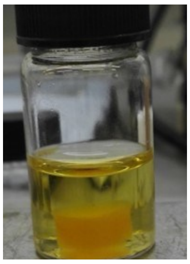

Figure S1: Pd uptake

Table S1: Absorption of standard solution of $PdCl_2$

Sample	Conc. Of $PdCl_2$ [mM]	Absorbance (λ = 425 nm)
1	0.7083	0.20
2	0.3541	0.13
3	0.2361	0.11
4	0.1180	0.083
5	0.0708	0.069
6	0.0472	0.067
7	0.0236	0.059

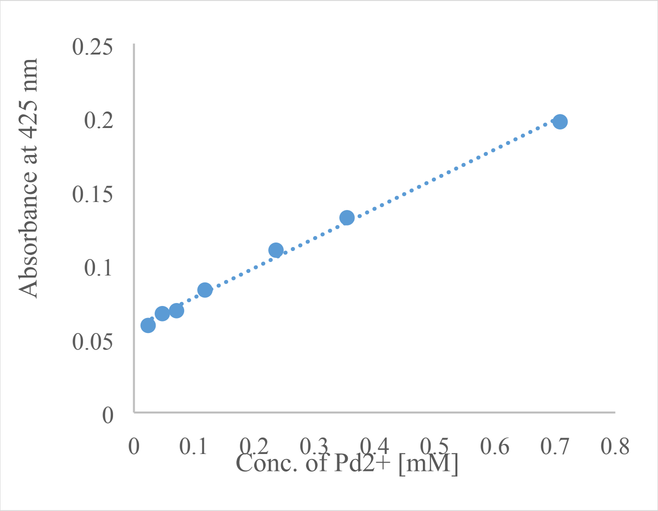

Figure S2: Calibration curve for Pd^{2+} ions.

Table S2: Uptake of $PdCl_2$ at room temperature (3 mL of 4.89 mM $PdCl_2$)

t [h]	Absorbance (λ = 425 nm)	Amount of Pd in gel [μmol]
1	0.894	2.18
2	0.795	3.66
3	0.686	5.28
4	0.661	5.66
21	0.582	6.84
24	0.578	6.90
48	0.530	7.61
72	0.504	8.00

Table S3: Uptake of $PdCl_2$ at 50 °C (3 mL of 4.39 mM $PdCl_2$)

t [h]	Absorbance (λ = 425 nm)	Amount of Pd in gel [µmol]
0.5	0.740	3.00
1	0.679	3.91
2	0.600	5.09
4	0.542	5.96
6	0.506	6.49
24	0.453	7.28
48	0.463	7.13

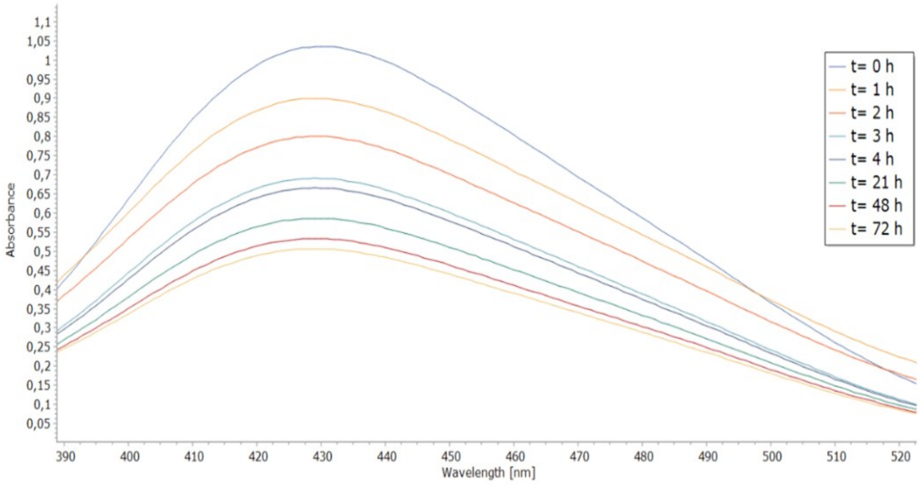

Figure S3: UV-Vis spectra of $PdCl_2$ solution after interaction with DBS-CONHNH$_2$-Agarose hybrid hydrogel at room temperature.

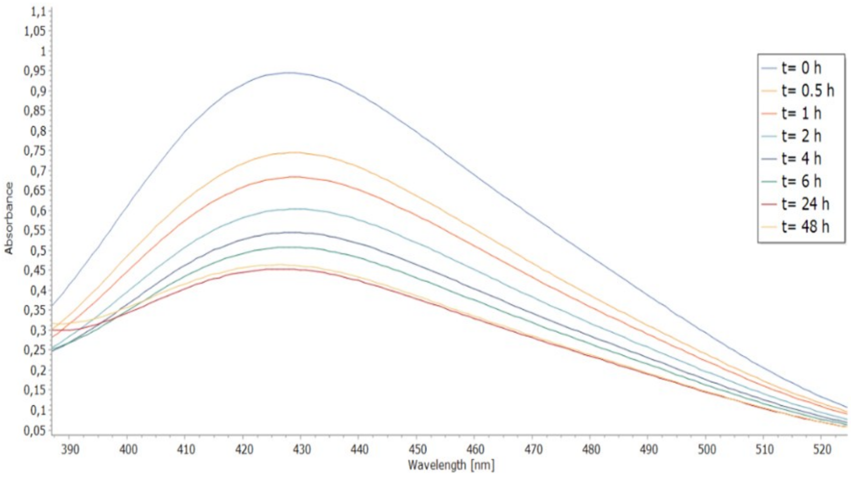

Figure S4: UV-Vis spectra of $PdCl_2$ solution after interaction with DBS-CONHNH$_2$-Agarose hybrid hydrogel at 50 °C.

4. Rheological Studies

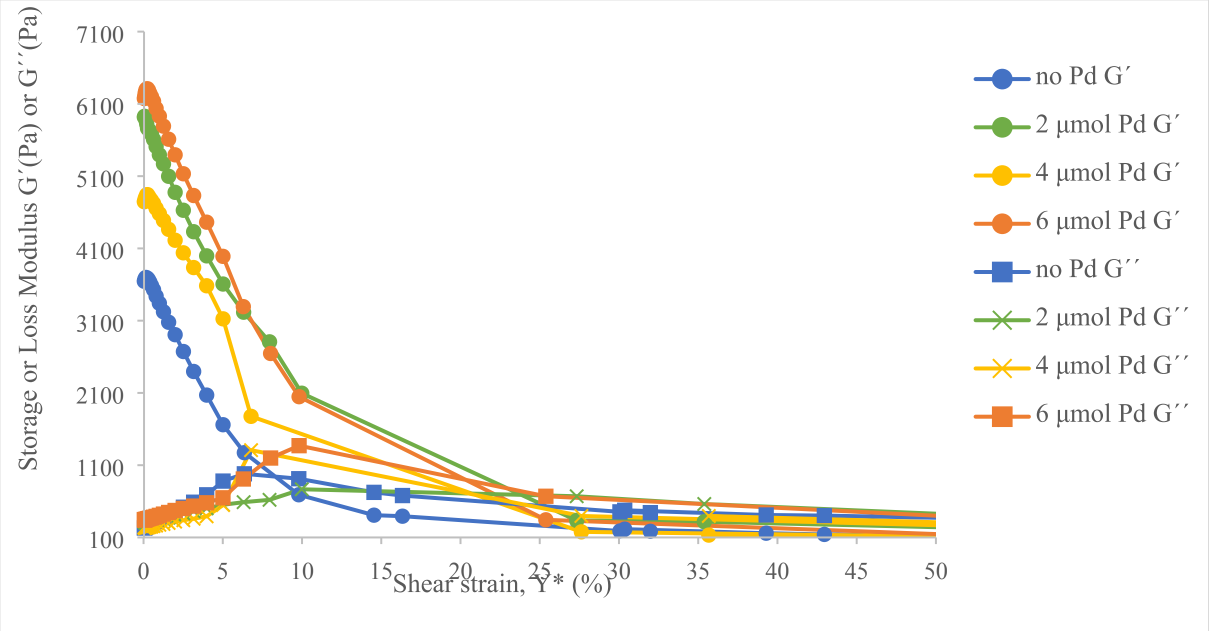

Figure S5: Strain amplitude dependence of the storage modulus (G') and loss modulus (G'') for hybrid hydrogel (0.82% wt/vol total) in the absence and presence of PdNPs. Frequency = 1 Hz.

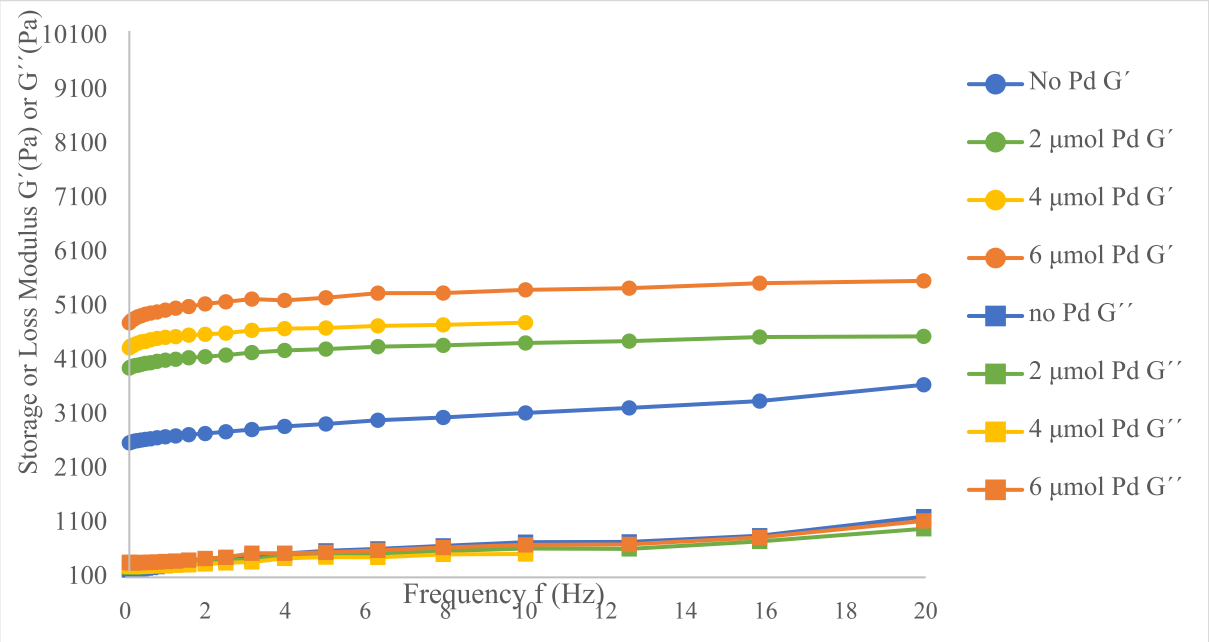

Figure S6: Frequency dependence of the storage modulus (G') and loss modulus (G'') for hybrid hydrogel (0.82% wt/vol total) in the absence and presence of PdNPs. Strain = 0.25%

5. General procedure for Suzuki cross-coupling reaction

Arylhalide (0.80 mmol), boronic acid (0.96 mmol) and K_2CO_3 (1.60 mmol) was dissolved in 4 mL of EtOH/H_2O mixture (3:1) in a reaction vial. The hybrid hydrogel containing approx. 8 µmol of Pd (1% mol) was added to the reaction mixture. The reaction vial was heated to 50 °C and left to react without stirring. Progress of the reaction was monitored by TLC. After the reaction was complete, product was extracted with diethyl ether and washed with 1M NaOH and water. The organic phase was dried over $MgSO_4$ and solvent was evaporated under reduced pressure to give the desired product.

6. Catalyst recyclability studies- Suzuki coupling

The coupling reaction between 4-iodotoluene and phenylboronic acid was carried out as described in the general procedure for Suzuki Cross-Coupling. After the reaction was finished, product was extracted with diethyl ether and gel was further washed with water. The gel was then directly used in another reaction.

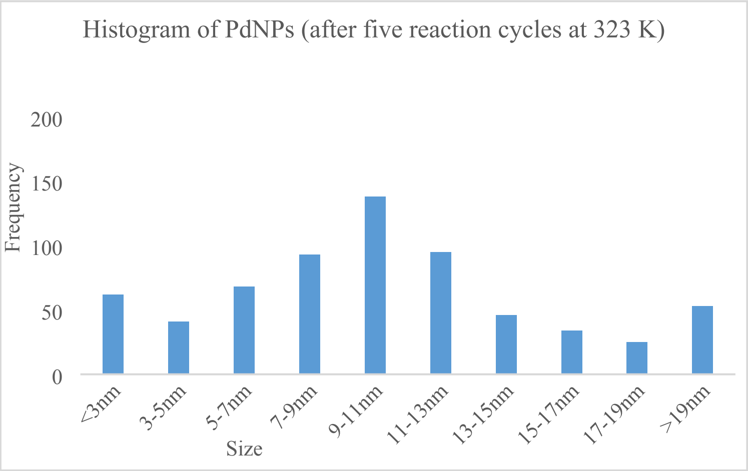

Figure S7: Historgram of PdNPs size distribution.

To test the effect of heating on nanoparticles in the absence of reaction, a sample of gel containing PdNPs was heated at 50°C and then imaged by TEM. A small amount of evidence of nanoparticle aggregation and enlargement was observed.

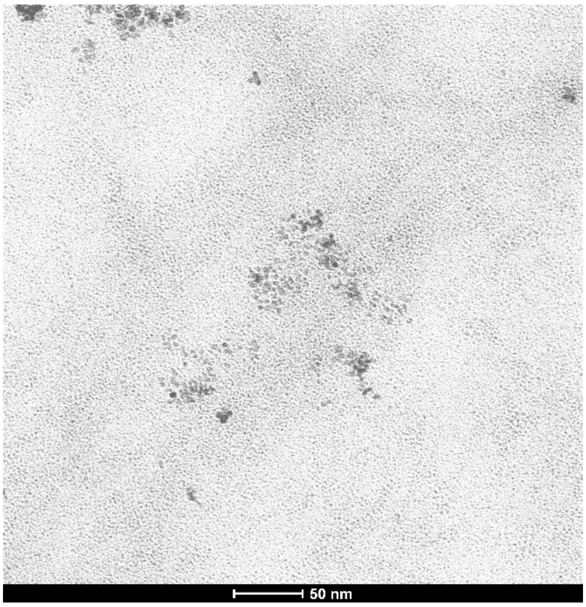

Figure S8. TEM image of sample of hybrid gel containing PdNPs heated at 50°C for 18 h prior to cooling and sample preparation.

7. Hot filtration test

To perform the hot filtration test, the reaction between 4-iodotoluene (0.6 mmol) and phenylboronic acid (0.72 mmol) was performed according to the general procedure for Suzuki coupling. After 24 hours (complete conversion- monitored by TLC) the hot reaction mixture was filtered to another vial using nylon syringe filter (0.22 µm). To this solution, 4-iodoanisole (0.6 mmol), phenylboronic acid (0.72 mmol) and K_2CO_3 (1.2 mmol) was added and another Suzuki coupling was performed (50 °C, no stirring). After 24 hours products were extracted with diethyl ether and washed with 1M NaOH and water. The crude reaction mixture was analysed by ^1H NMR to determine reaction conversion.

8. Suzuki cross-coupling in a syringe

A known quantity of DBS-$CONHNH_2$ (usually 2.0 mg, 4.47 µmol) was weighed into a 2.5 mL sample vial and 0.65 mL deionised water was added. The vial was then sonicated to disperse the solid. The mixture was heated with shaking until the gelator completely dissolved. The hydrosol was transferred into a 3 mL syringe where small amount of cotton was placed at the bottom. The hydrosol was allowed to cool down at room temperature as the hydrogel formed. The cork of the syringe was temporarily blocked by parafilm. The solution of $PdCl_2$ was put on the top of a gel and left to interact for 48 hours. After that time, parafilm was removed and solution was left to diffuse through the gel. The gel was further washed several times with deionised water. In order to perform Suzuki coupling, reagents were put on the top of the gel in desired solvent (EtOH/H_2O mixture or PEG 200) and the syringe was placed into

the incubator set to 50 °C. After diffusion through the gel, products were collected in a vial. Gel was further washed several times with desired solvent (EtOH/H_2O mixture or PEG 200) to fully extract the products from the gel. The extraction and washing steps were the same as in the general procedure.

9. Pd scavenging experiment in flow-through device

A known quantity of DBS-CONHNH$_2$ (3.1 mg) was weighed into a 2.5 mL sample vial and 0.75 mL deionised water was added. The vial was then sonicated to disperse the solid. The mixture was heated with shaking until the gelator completely dissolved. The hydrosol was transferred into a 1 mL syringe where small amount of cotton was placed at the bottom. The hydrosol was allowed to cool down at room temperature as the hydrogel formed. The syringe was placed in the incubator set to 50 °C. Solution of $PdCl_2$ (0.7 mL, 2.86 mM) was put in portions on the top of the gel and let to diffuse through gel. After complete filtration (20 min), the gel was further washed with 0.8 mL of deionised water. Collected colourless filtrate mixed with 3 mL of EtOH was directly used as a solvent for Suzuki coupling between 4-iodotoluene (0.40 mmol) and phenylboronic acid (0.41 mmol) using K_2CO_3 (0.80 mmol) as a base. The reaction was kept without stirring at 50 °C for 24 hours. The crude reaction mixture was analysed by ^{1}H NMR.

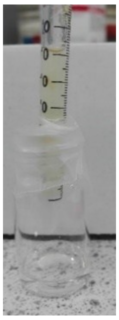

Figure S9: Pd scavenging experiments – filtration over gel in the syringe

10. Characterization data of the products

4-Methylbiphenyl (2a)

White solid. ^{1}H NMR ($CDCl_3$, 400 MHz, 293 K): δ = 7.62 – 7.57 (m, 2H), 7.54 – 7.48 (m, 2H), 7.48 – 7.41 (m, 2H), 7.37 – 7.30 (m, 1H), 7.30 – 7.24 (m, 2H), 2.41 (s, 3H) ppm. ^{13}C NMR ($CDCl_3$, 100 MHz, 293 K): δ = 141.3, 138.5, 137.1, 129.6, 128.8, 127.3, 127.1 (2x), 21.2 ppm. The recorded spectroscopic data correlate with those reported in literature.[2]

4-Methoxybiphenyl (2b)

White solid. ^{1}H NMR ($CDCl_3$, 400 MHz, 293 K): δ = 7.60 – 7.52 (m, 4H), 7.46 – 7.40 (m, 2H), 7.35 – 7.29 (m, 1H), 7.02 – 6.97 (m, 2H), 3.86 (s, 3H) ppm. ^{13}C NMR ($CDCl_3$, 100 MHz, 293 K): δ = 159.3, 140.9,

133.9, 128.9, 128.3, 126.9, 126.8, 114.3, 55.5 ppm. The recorded spectroscopic data correlate with those reported in literature.[2]

3,4-Dimethoxybiphenyl (2c)

White solid. ^{1}H NMR ($CDCl_3$, 400 MHz, 293 K): δ = 7.58 – 7.53 (m, 2H), 7.45 – 7.39 (m, 2H), 7.34 – 7.28 (m, 1H), 7.17 – 7.10 (m, 2H), 6.94 (d, 1H, *J* = 8.3 Hz), 3.95 (s, 3H), 3.92 (s, 3H) ppm. ^{13}C NMR ($CDCl_3$, 100 MHz, 293 K): δ = 149.2, 148.7, 141.2, 134.3, 128.9, 127.0 (2x), 119.5, 111.5, 110.5, 56.1, 56.0 ppm. The recorded spectroscopic data correlate with those reported in literature.[2]

4-Aminobiphenyl (2d)

Brown solid. ^{1}H NMR ($CDCl_3$, 400 MHz, 293 K): δ = 7.61 – 7.55 (m, 2H), 7.48 – 7.39 (m, 4H), 7.34 – 7.27 (m, 1H), 6.80 – 6.73 (m, 2H), 3.72 (s, 2H) ppm. ^{13}C NMR ($CDCl_3$, 100 MHz, 293 K): δ = 145.9, 141.3, 131.7, 128.8, 128.1, 126.5, 126.4, 115.5 ppm. The recorded spectroscopic data correlate with those reported in literature.[3]

4-Chlorobiphenyl (2e)

White solid. ^{1}H NMR ($CDCl_3$, 400 MHz, 293 K): δ = 7.61 – 7.51 (m, 4H), 7.50 – 7.35 (m, 5H) ppm. ^{13}C NMR ($CDCl_3$, 101 MHz, 293 K): δ = 140.0, 139.7, 133.4, 128.9, 128.4, 127.6, 127.0 ppm. The recorded spectroscopic data correlate with those reported in literature.[2]

3-Chlorobiphenyl (2f)

White solid. ^{1}H NMR ($CDCl_3$, 400 MHz, 293 K): δ = 7.68 – 7.58 (m, 3H), 7.55 – 7.46 (m, 3H), 7.46 – 7.35 (m, 3H) ppm. ^{13}C NMR ($CDCl_3$, 101 MHz, 293 K): δ = 143.1, 139.9, 134.7, 130.1, 129.0, 127.9, 127.4, 127.3, 127.2, 125.4 ppm. The recorded spectroscopic data correlate with those reported in literature.[4]

4-Fluorobiphenyl (2g)

White solid. ^{1}H NMR ($CDCl_3$, 400 MHz, 293 K): δ = 7.64 – 7.52 (m, 4H), 7.49 – 7.41 (m, 2H), 7.39 – 7.34 (m, 1H), 7.18 – 7.10 (m, 2H) ppm. ^{13}C NMR ($CDCl_3$, 101 MHz, 293 K): δ = 162.5 (d, $J_{C\text{-}F}$ = 246.2 Hz), 140.4, 137.5 (d, $J_{C\text{-}F}$ = 3.2 Hz), 129.0, 128.8 (d, $J_{C\text{-}F}$ = 8.0 Hz), 127.4, 127.2, 115.8 (d, $J_{C\text{-}F}$ = 21.4 Hz) ppm. The recorded spectroscopic data correlate with those reported in literature.[2]

4-Acetylbiphenyl (2h)

White solid. ^{1}H NMR ($CDCl_3$, 400 MHz, 293 K): δ = 8.03 (d, 1H, *J* = 8.2 Hz), 7.65 (dd, 2H, *J* = 15.9, 7.6 Hz), 7.52 – 7.34(m, 2H), 2.63 (s, 3H) ppm. ^{13}C NMR ($CDCl_3$, 101 MHz, 293 K): δ = 197.7, 145.7, 139.8, 135.8, 129.0, 128.9, 128.3, 127.3, 127.2, 26.7 ppm. The recorded spectroscopic data correlate with those reported in literature.[2]

4-Phenylbenzoic acid (2i)

White solid. ^{1}H NMR (DMSO-d_6, 400 MHz, 293 K): δ = 12.99 (s, 1H), 8.03 – 7.97 (m, 2H), 7.76 – 7.71 (m, 2H), 7.70 – 7.64 (m, 2H), 7.48 – 7.40 (m, 2H), 7.40 – 7.33(m, 1H) ppm. ^{13}C NMR (DMSO-d_6, 101 MHz, 293 K): δ = 167.7, 144.9, 139.6, 130.5, 130.2, 129.6, 128.8, 127.5, 127.3 ppm. The recorded spectroscopic data correlate with those reported in literature.[5]

4-Nitrobiphenyl (2j)

Yellow-white solid. ^{1}H NMR ($CDCl_3$, 400 MHz, 293 K): δ = 8.32 – 8.26 (m, 2H), 7.75 – 7.70 (m, 2H), 7.64 – 7.59 (m, 2H), 7.52 – 7.41 (m, 3H) ppm. ^{13}C NMR ($CDCl_3$, 101 MHz, 293 K): δ = 147.7, 147.2, 138.9, 129.3, 129.0, 127.9, 127.5, 124.2 ppm. The recorded spectroscopic data correlate with those reported in literature.[2]

2-Phenylthiophene (2k)

White solid. ^{1}H NMR ($CDCl_3$, 400 MHz, 293 K): δ = 7.73 – 7.59 (m, 2H), 7.52 – 7.29 (m, 5H), 7.17 – 7.09(m, 1H) ppm. ^{13}C NMR ($CDCl_3$, 101 MHz, 293 K): δ = 144.6, 134.6, 129.1, 128.2, 127.6, 126.1, 125.0, 123.3 ppm. The recorded spectroscopic data correlate with those reported in literature.[6]

3′,4′,5′,6′-Tetrafluoro-1,1′,2′,1″-terphenyl (2l)

White solid. ^{1}H NMR ($CDCl_3$, 400 MHz, 293 K): δ = 7.24 – 7.16 (m, 6H), 7.06 – 6.98 (m, 4H) ppm. ^{13}C NMR ($CDCl_3$, 101 MHz, 293 K): δ = 145.0 (m, $J_{C\text{-}F}$ = 245.5 Hz, 1C), 140.1 (m, $J_{C\text{-}F}$ = 256.0 Hz, 1C), 131.5, 130.8, 128.2, 128.1, 125.6 (m, 1C) ppm. The recorded spectroscopic data correlate with those reported in literature.[7]

Biphenyl (2m)

White solid. ^{1}H NMR ($CDCl_3$, 400 MHz, 293 K): δ = 7.69 – 7.64 (m, 4H), 7.53 – 7.46 (m, 4H), 7.43 – 7.37 (m, 2H) ppm. ^{13}C NMR ($CDCl_3$, 101 MHz, 293 K): δ = 141.4, 128.9, 127.4, 127.3 ppm. The recorded spectroscopic data correlate with those reported in literature.[2]

Biphenyl-4-carbaldehyde (2n)

White solid. ^{1}H NMR ($CDCl_3$, 400 MHz, 293 K): δ = 10.05 (s, 1H), 7.98 – 7.92 (m, 2H), 7.77 – 7.72 (m, 2H), 7.65 – 7.60 (m, 2H), 7.51 – 7.45 (m, 2H), 7.44 – 7.38 (m, 1H) ppm. ^{13}C NMR ($CDCl_3$, 101 MHz, 293 K): δ = 191.9, 147.2, 139.7, 135.2, 130.3, 129.0, 128.5, 127.7, 127.4 ppm. The recorded spectroscopic data correlate with those reported in literature.[2]

1-phenylnaphthalene (2o)

Colourless liquid. ^{1}H NMR ($CDCl_3$, 400 MHz, 293 K): δ = 7.99 – 7.87 (m, 3H), 7.60 – 7.43 (m, 9H) ppm. ^{13}C NMR ($CDCl_3$, 101 MHz, 293 K): δ = 141.0, 140.4, 134.0, 131.8, 130.3, 128.5, 127.8, 127.4, 127.1, 126.2, 126.0, 125.6 ppm. The recorded spectroscopic data correlate with those reported in literature.[8]

11. Copies of ^{1}H and ^{13}C NMR spectra

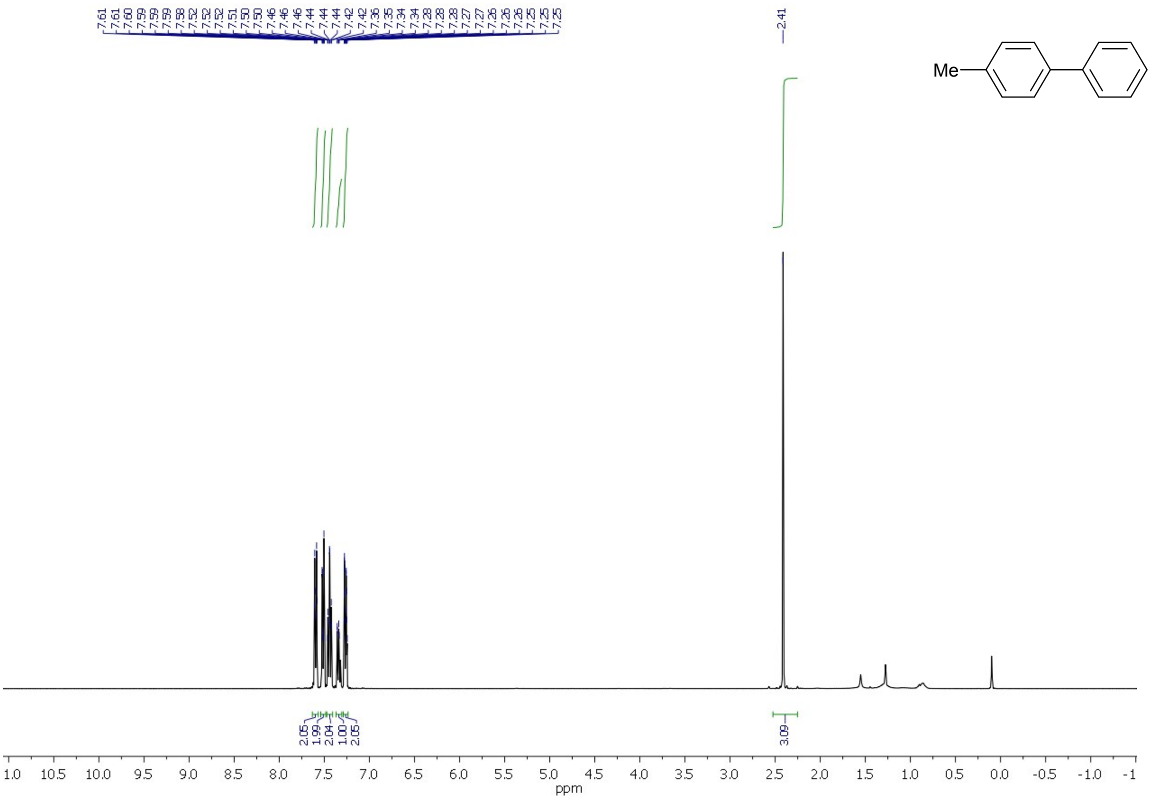

Figure S10: ^{1}H NMR of compound **2a** ($CDCl_3$, 400 MHz).

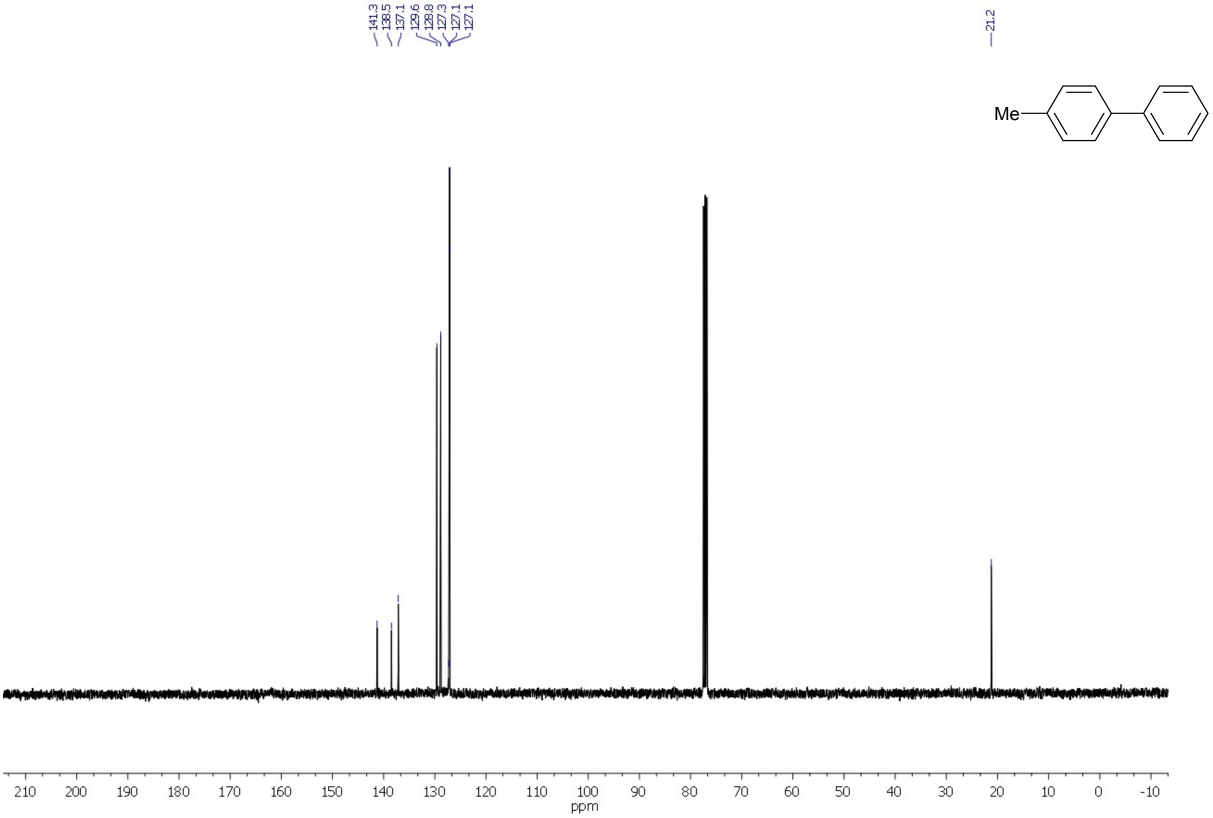

Figure S11: ^{13}C NMR of compound **2a** ($CDCl_3$, 101 MHz).

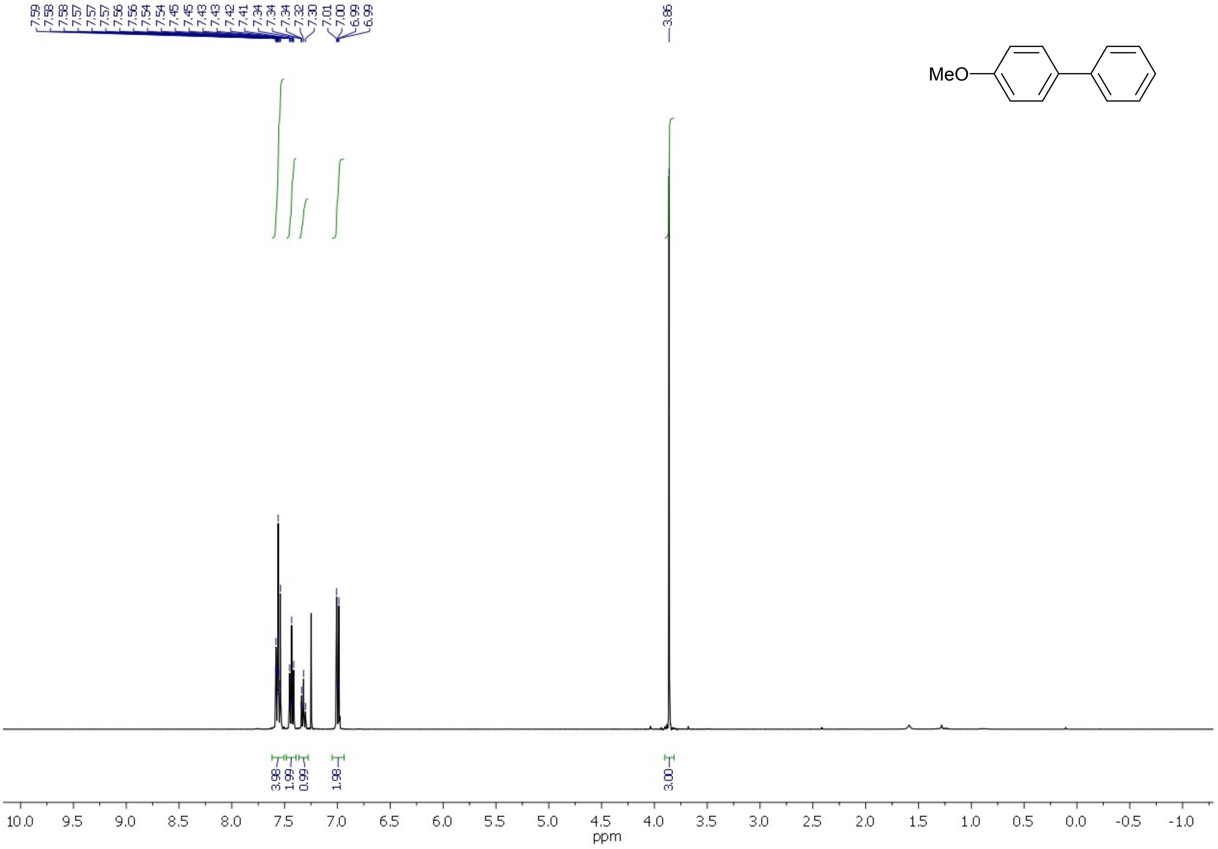

Figure S12: ^{1}H NMR of compound **2b** ($CDCl_3$, 400 MHz).

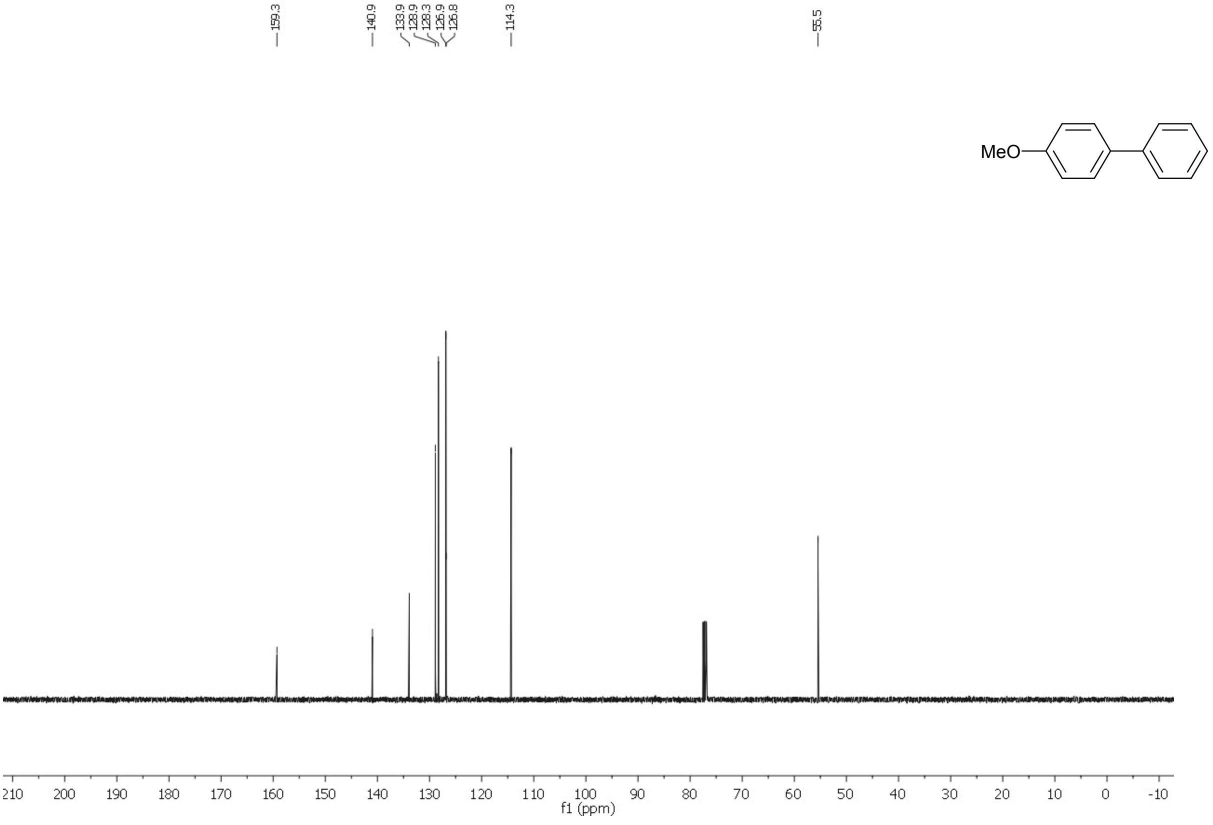

Figure S13: ^{13}C NMR of compound **2b** ($CDCl_3$, 101 MHz).

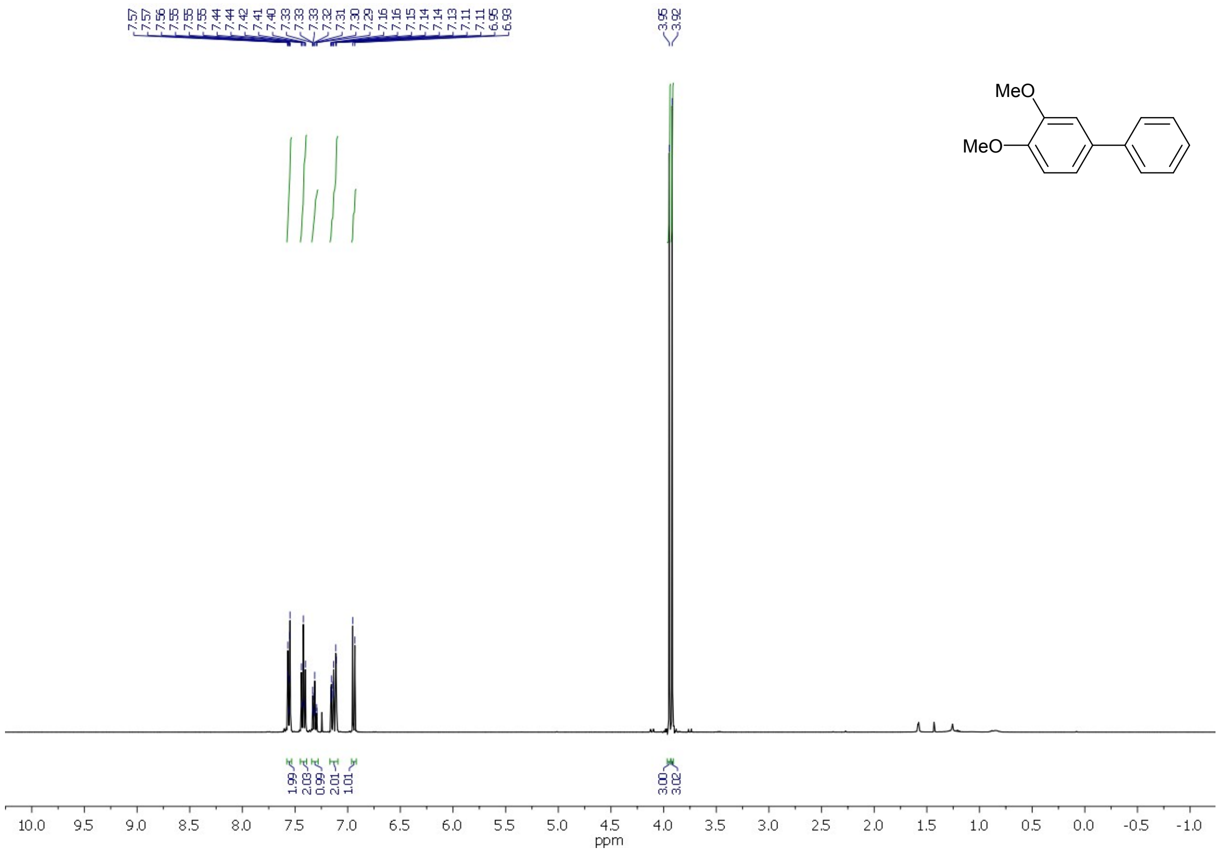

Figure S14: ^{1}H NMR of compound **2c** ($CDCl_3$, 400 MHz).

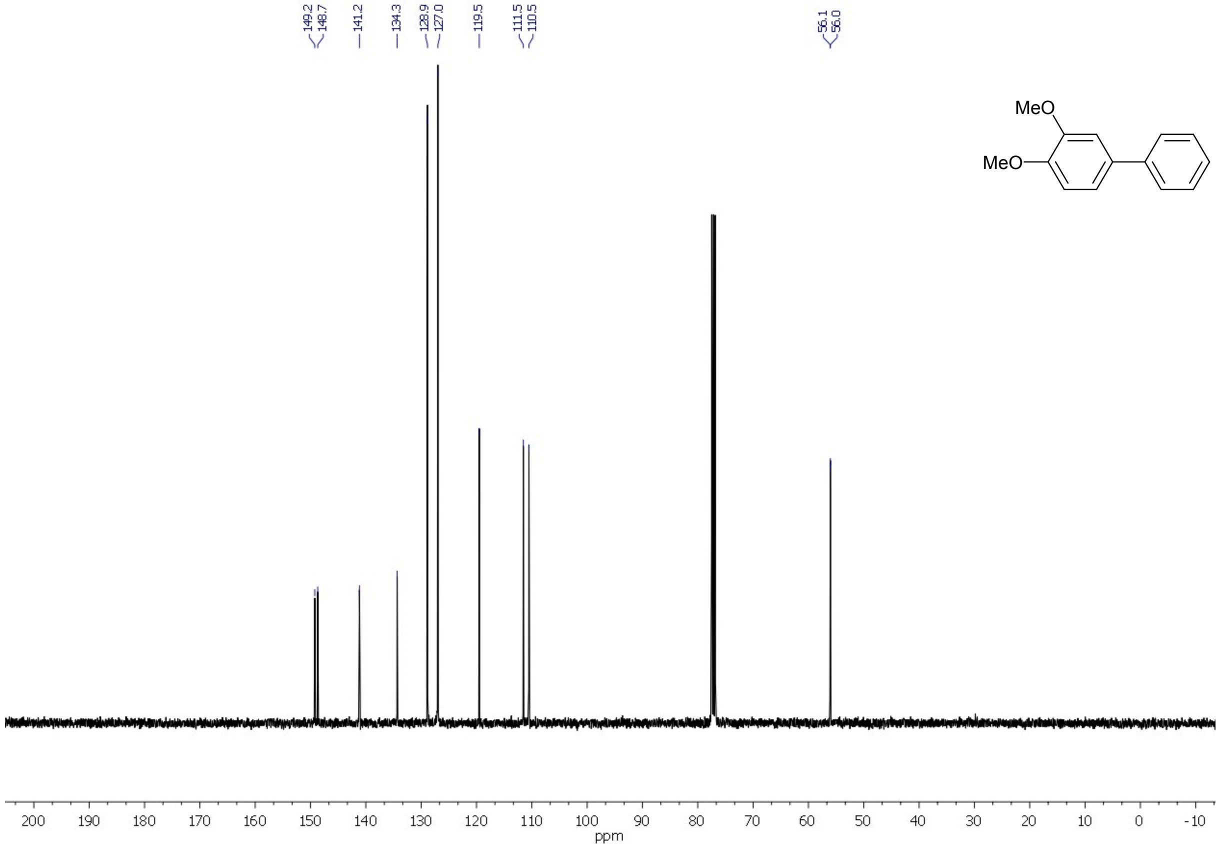

Figure S15: ^{13}C NMR of compound **2c** ($CDCl_3$, 101 MHz).

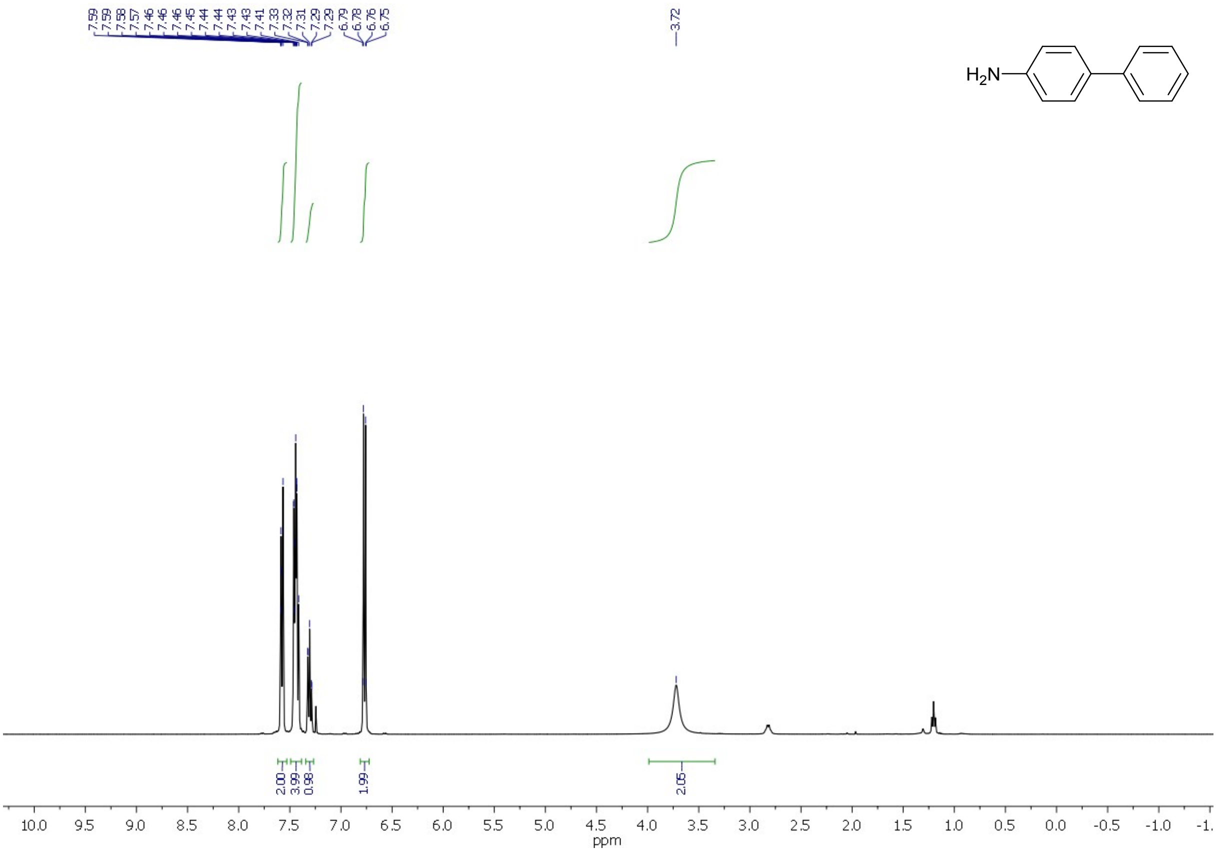

Figure S16: ^{1}H NMR of compound **2d** ($CDCl_3$, 400 MHz).

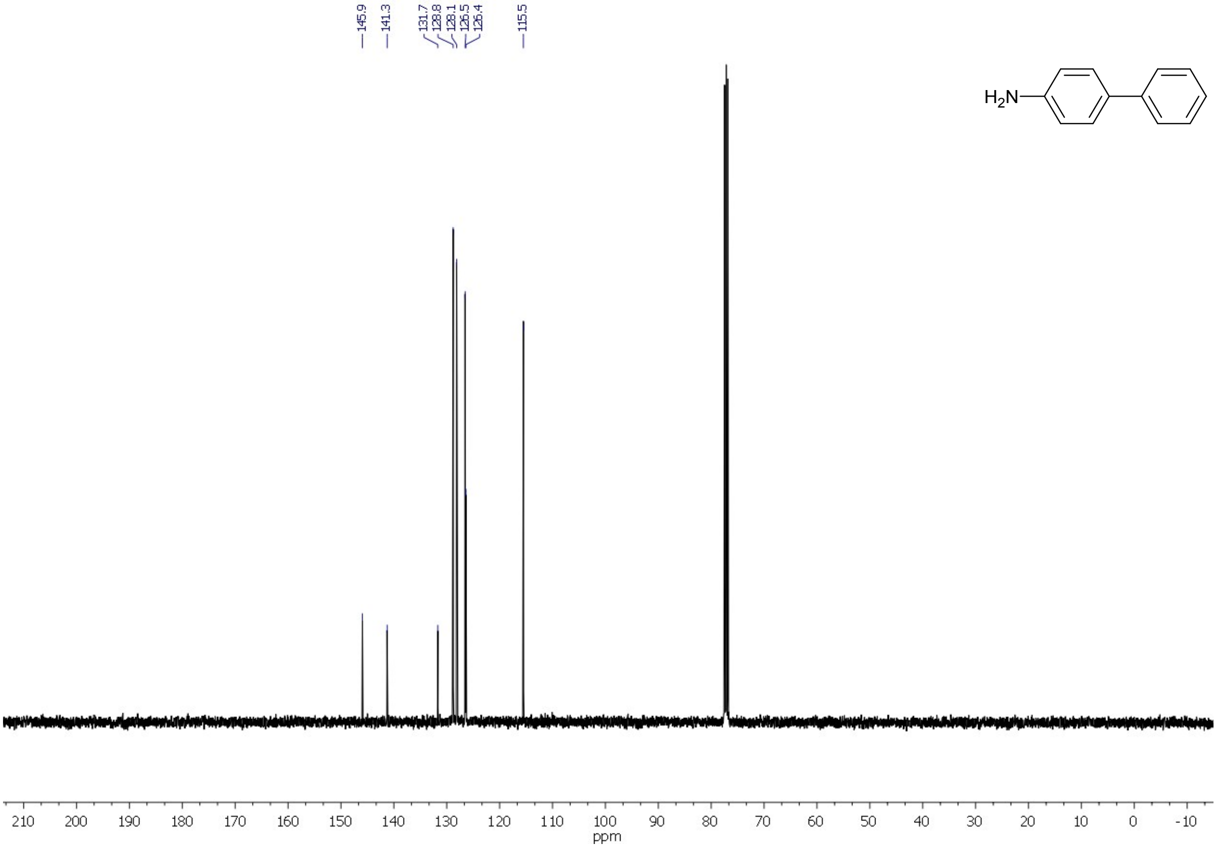

Figure S17: ^{13}C NMR of compound **2d** ($CDCl_3$, 101 MHz).

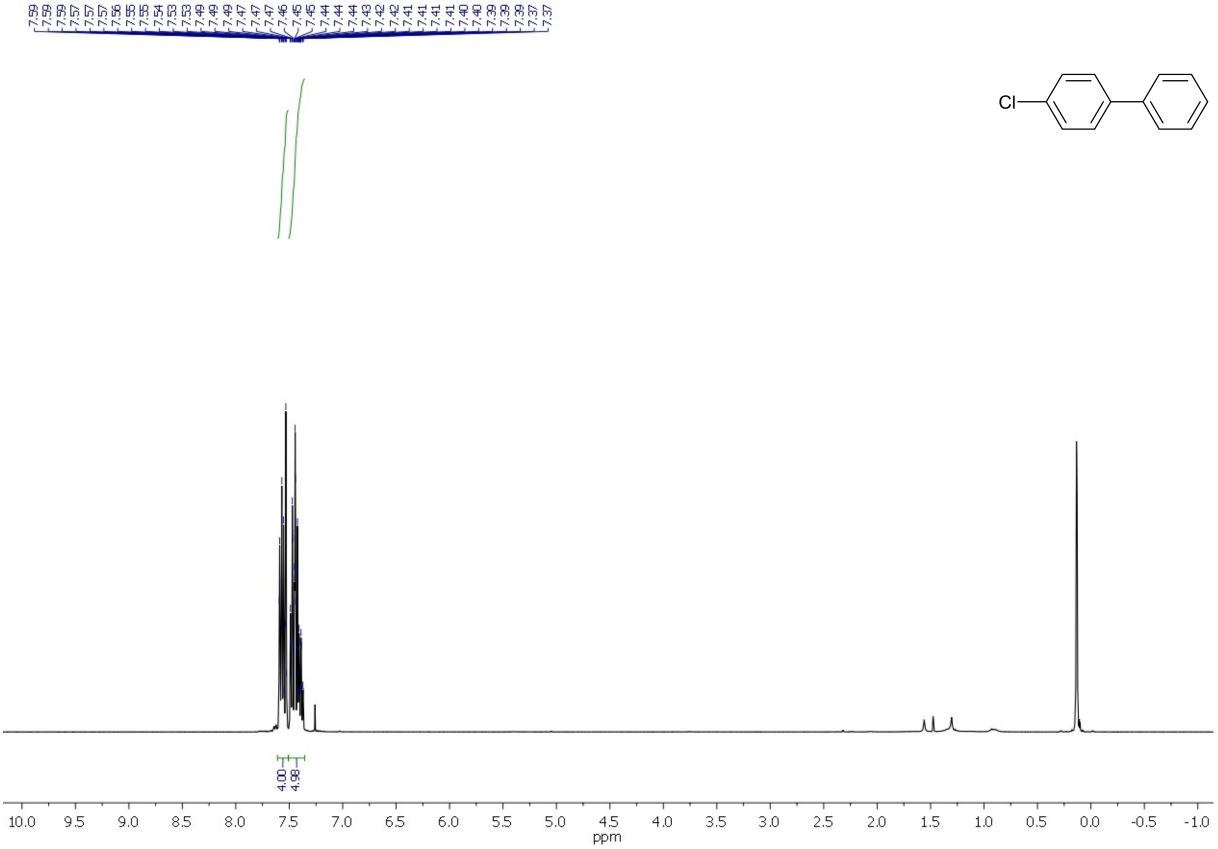

Figure S18: ^{1}H NMR of compound **2e** ($CDCl_3$, 400 MHz).

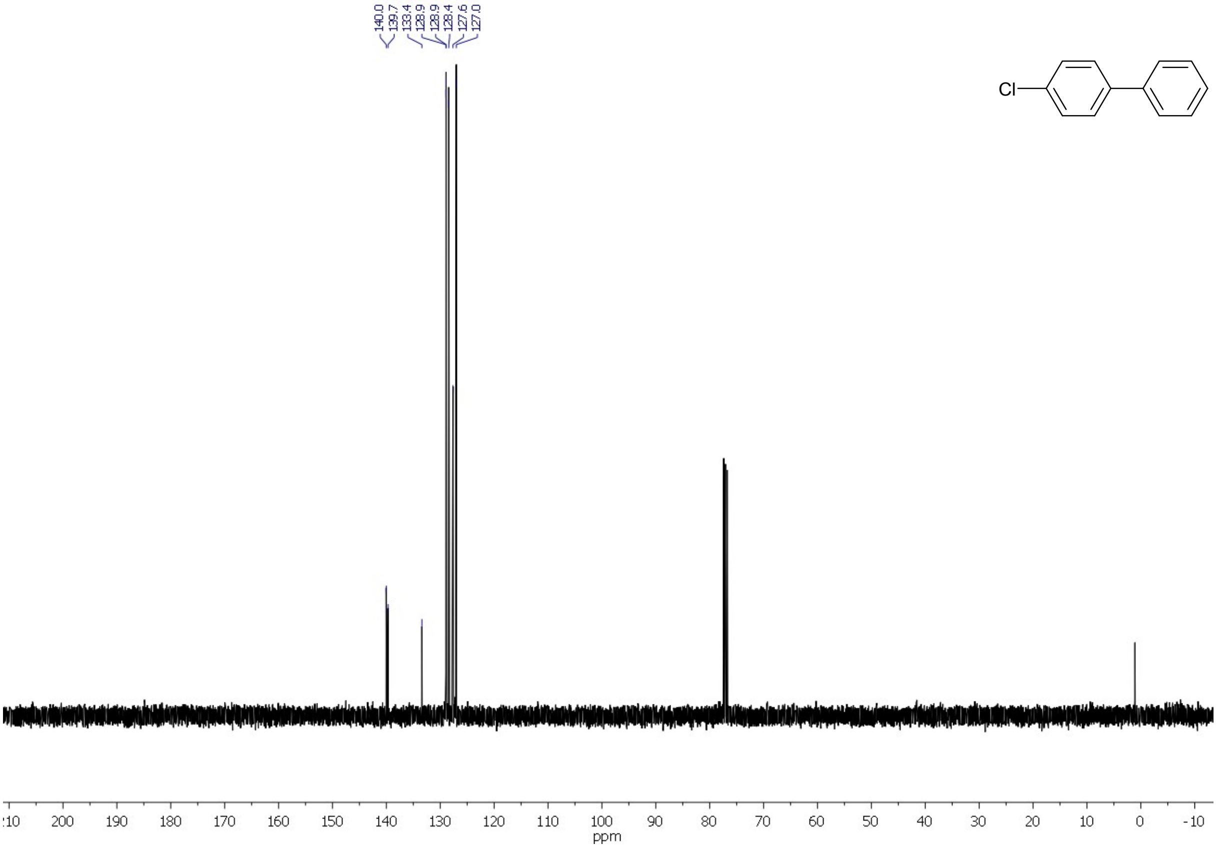

Figure S19: ^{13}C NMR of compound **2e** ($CDCl_3$, 101 MHz).

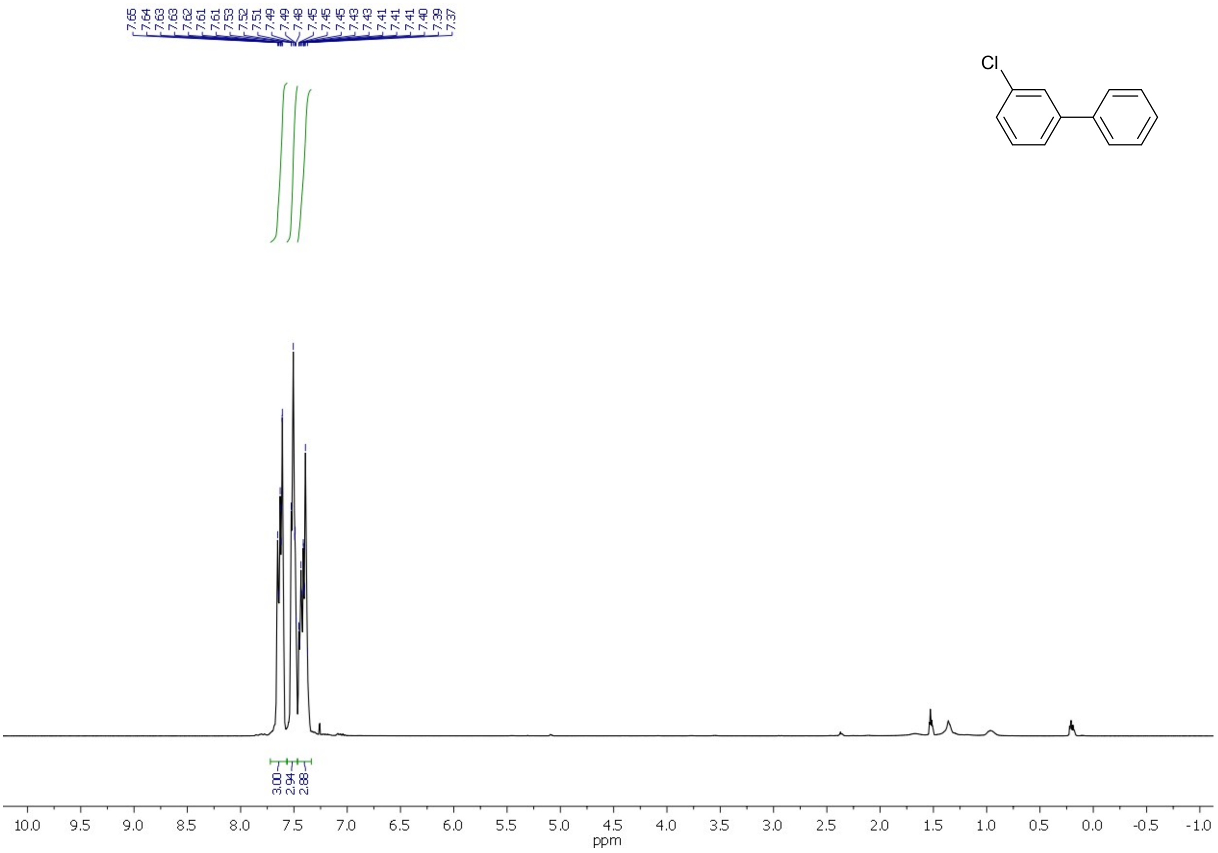

Figure S20: ^{1}H NMR of compound **2f** ($CDCl_3$, 400 MHz).

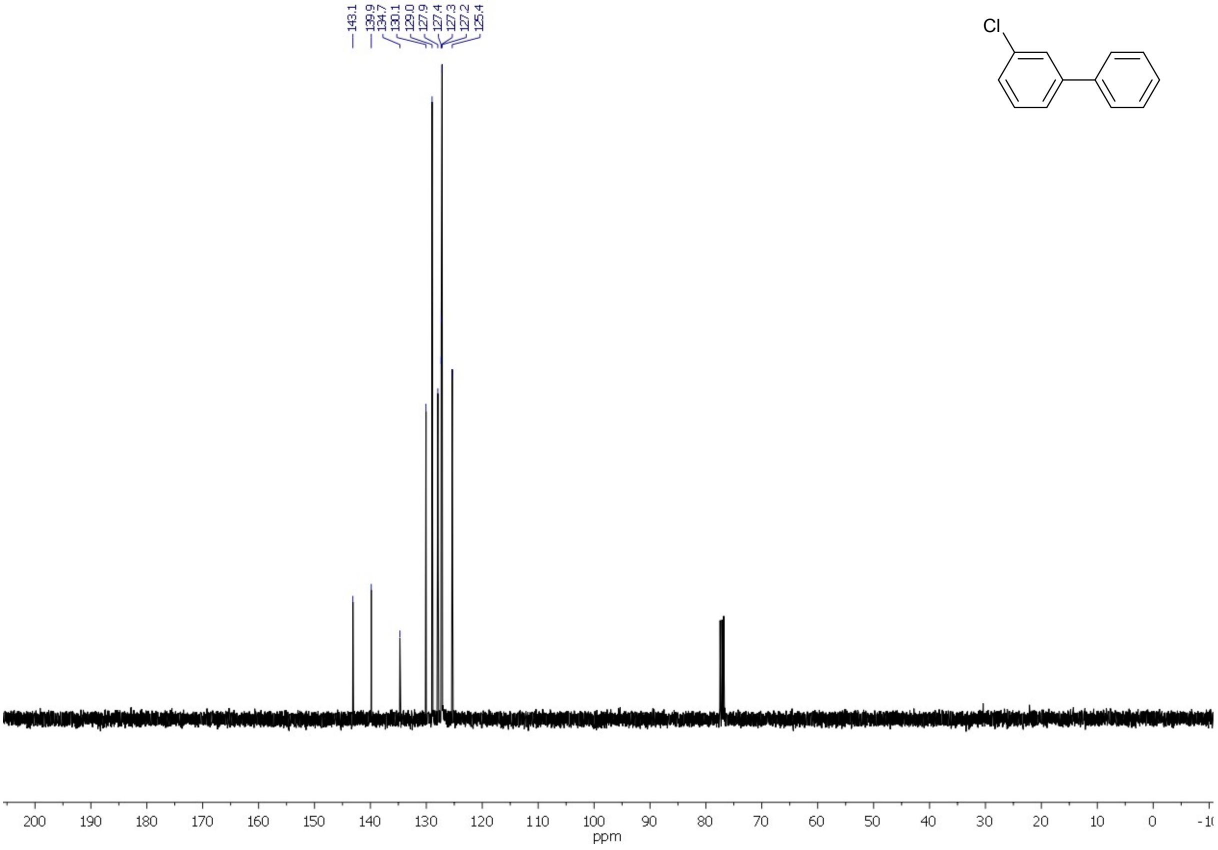

Figure S21: ^{13}C NMR of compound **2f** ($CDCl_3$, 101 MHz).

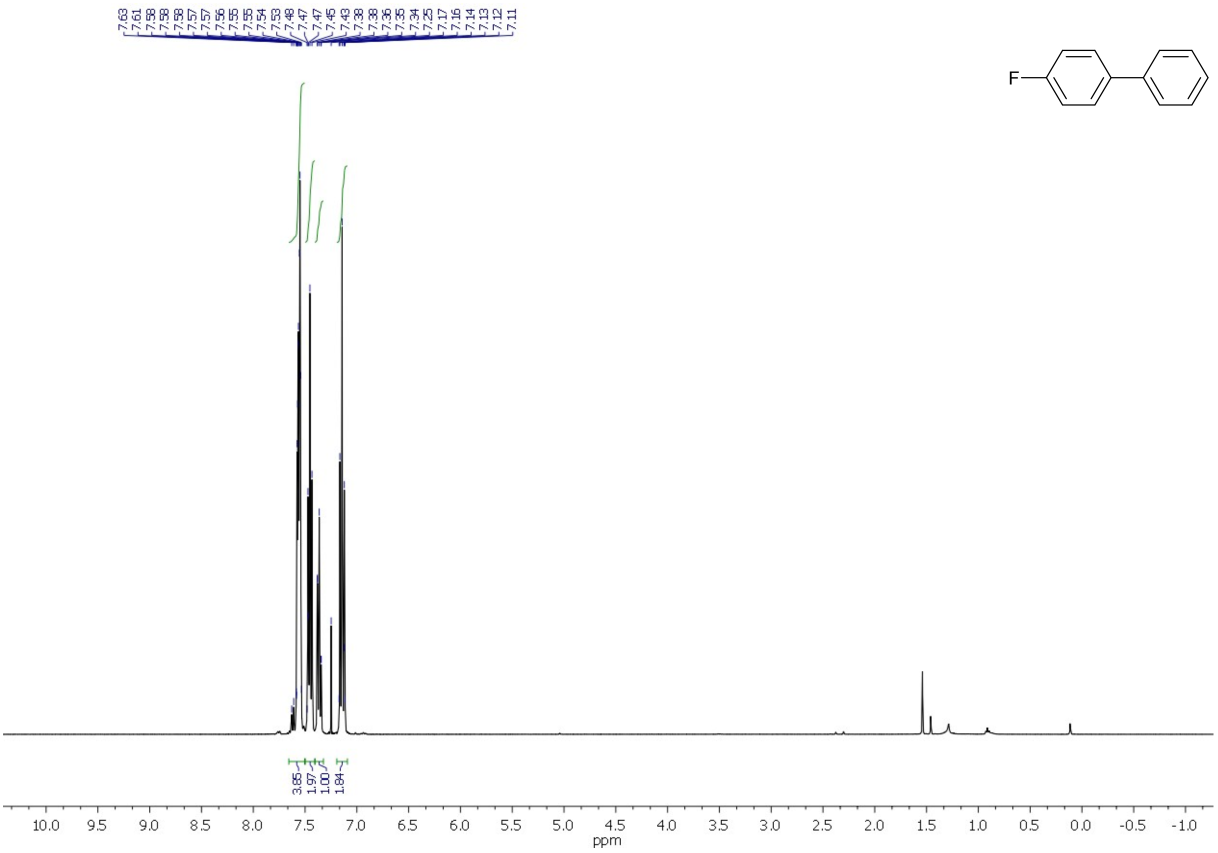

Figure S22: ^{1}H NMR of compound **2g** ($CDCl_3$, 400 MHz).

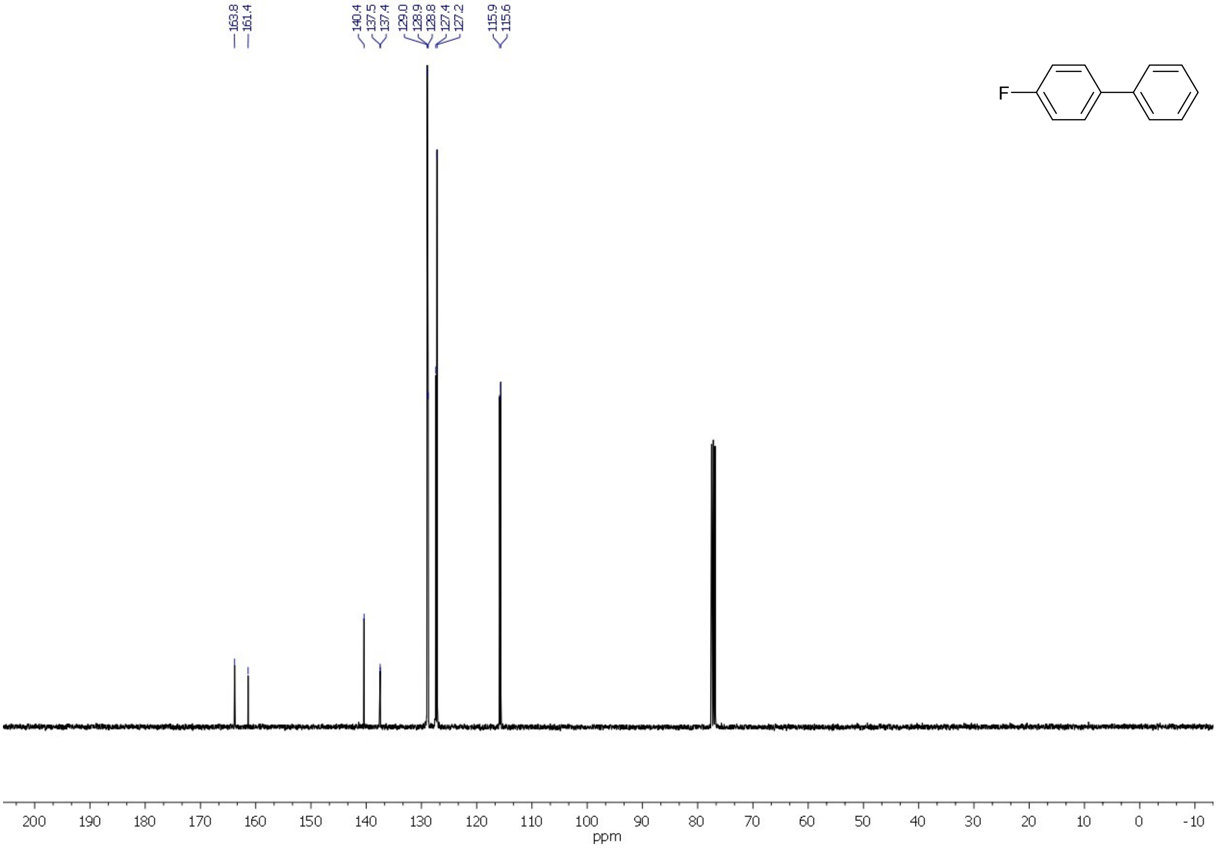

Figure S23: ^{13}C NMR of compound **2g** ($CDCl_3$, 101 MHz).

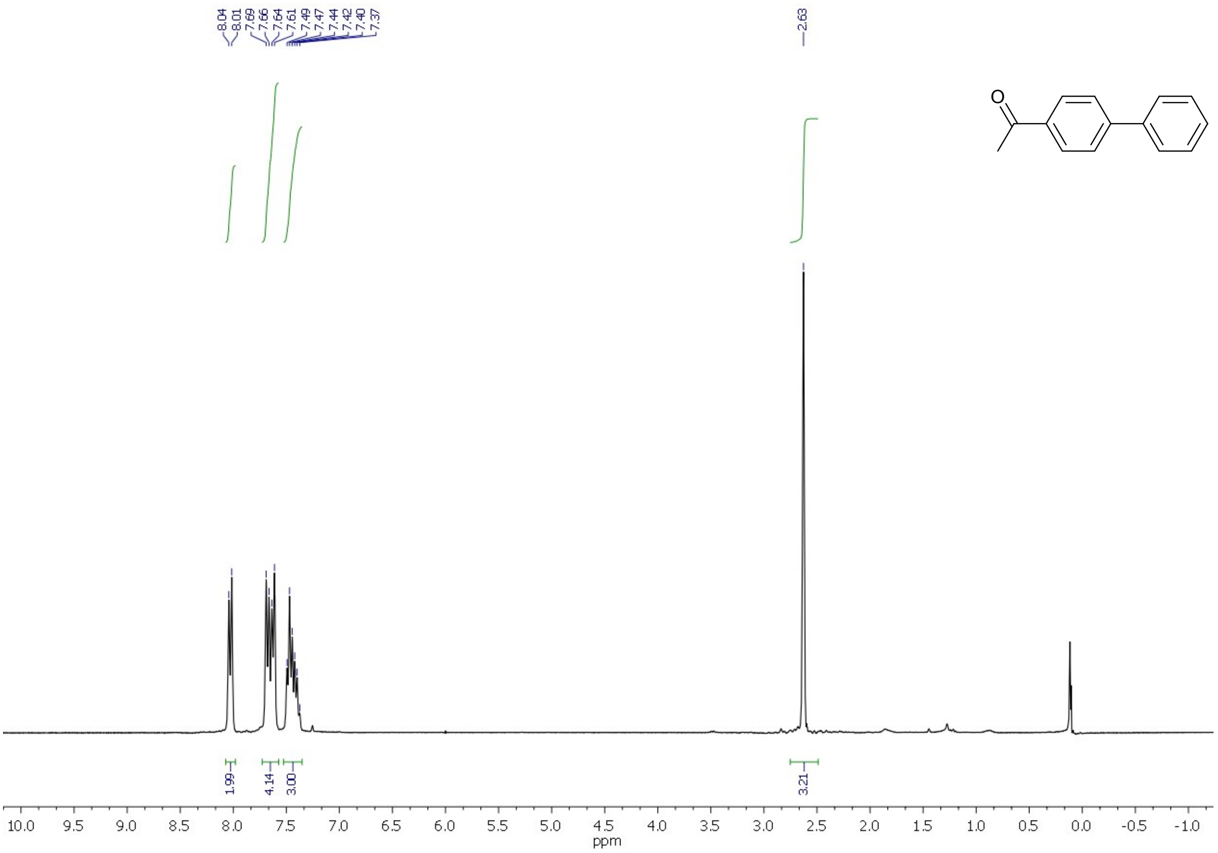

Figure S24: ^{1}H NMR of compound **2h** ($CDCl_3$, 400 MHz).

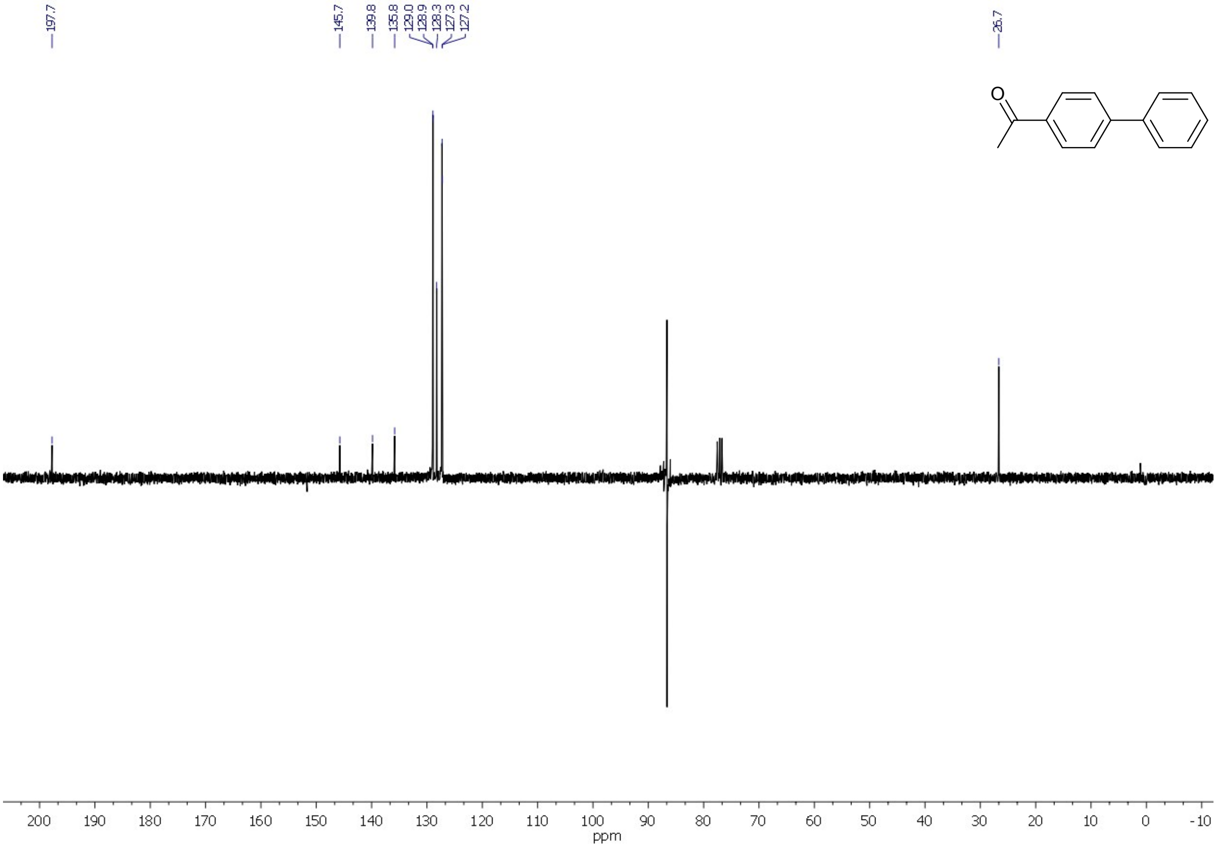

Figure S25: ^{13}C NMR of compound **2h** ($CDCl_3$, 101 MHz).

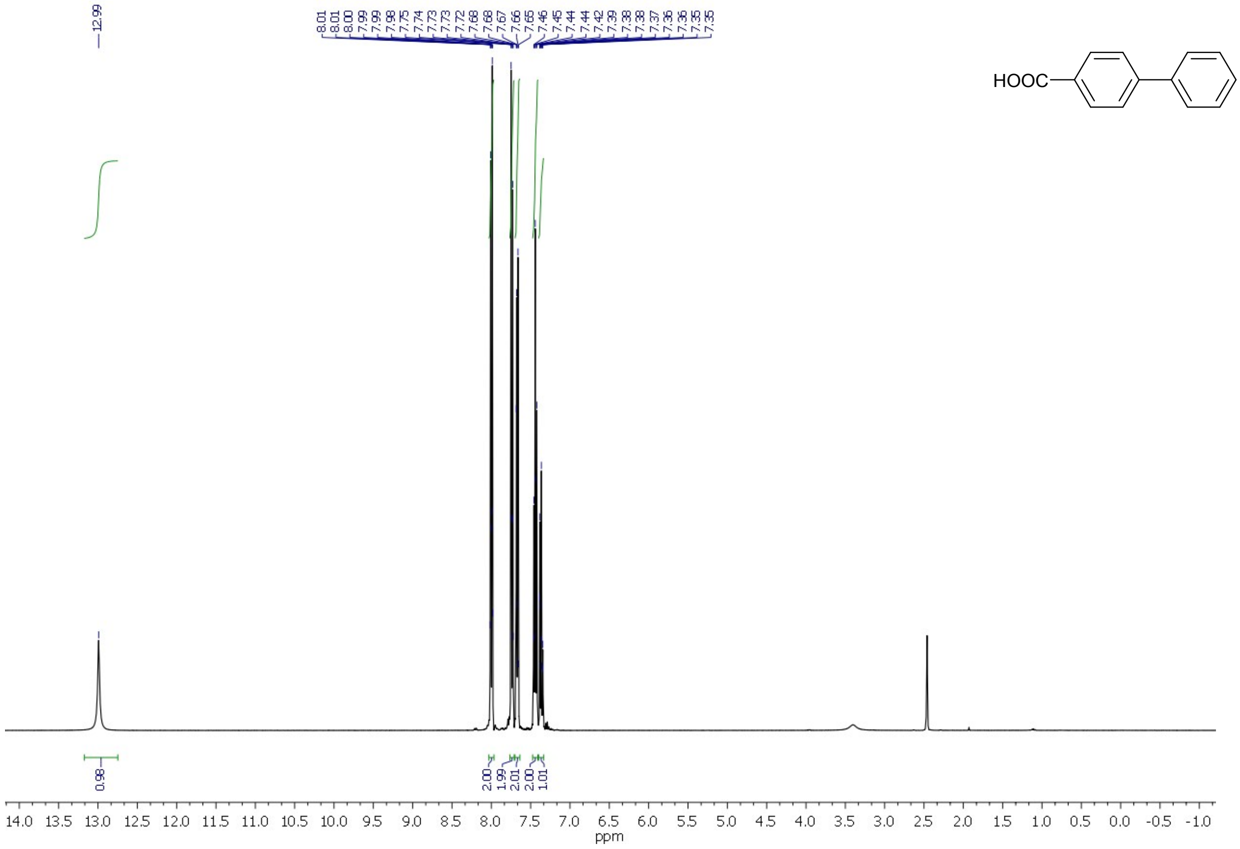

Figure S26: ^{1}H NMR of compound **2i** ($CDCl_3$, 400 MHz).

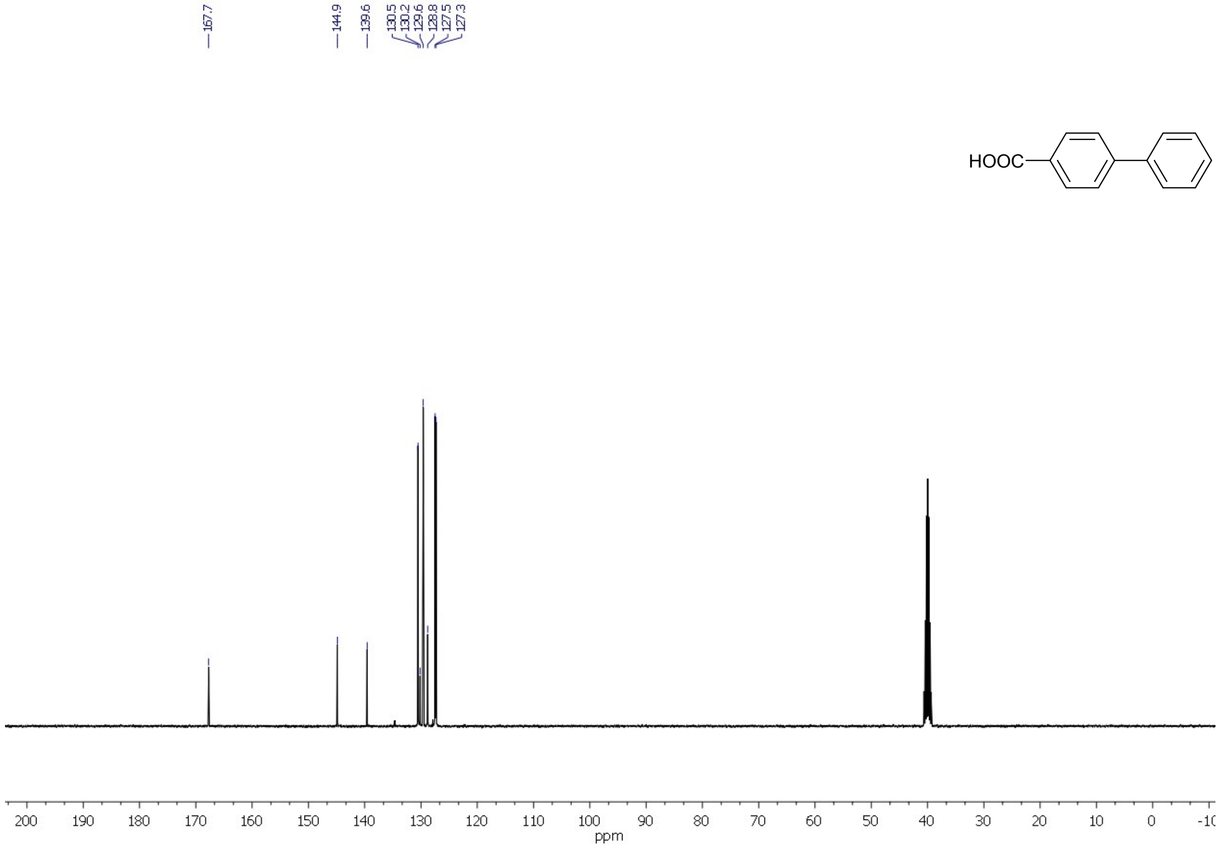

Figure S27: ^{13}C NMR of compound **2i** ($CDCl_3$, 101 MHz).

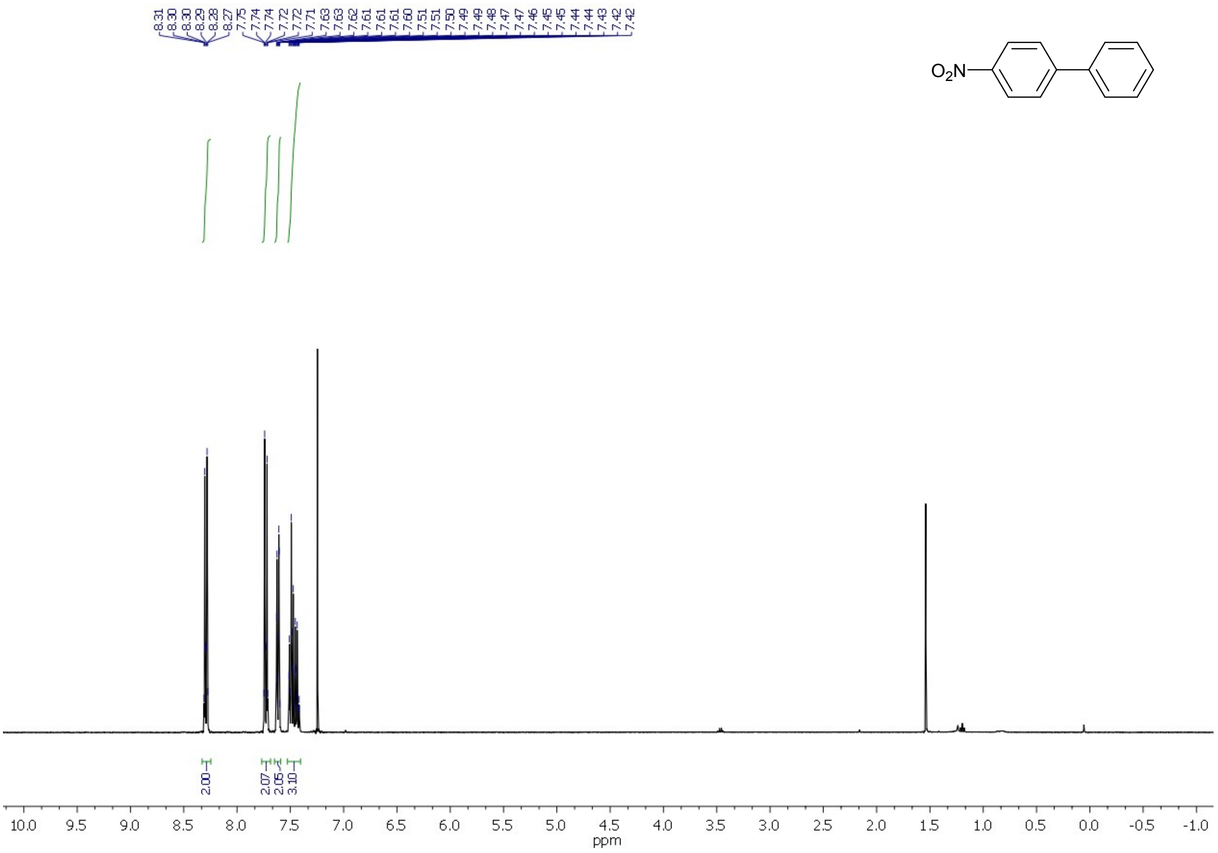

Figure S28: ^{1}H NMR of compound **2j** ($CDCl_3$, 400 MHz).

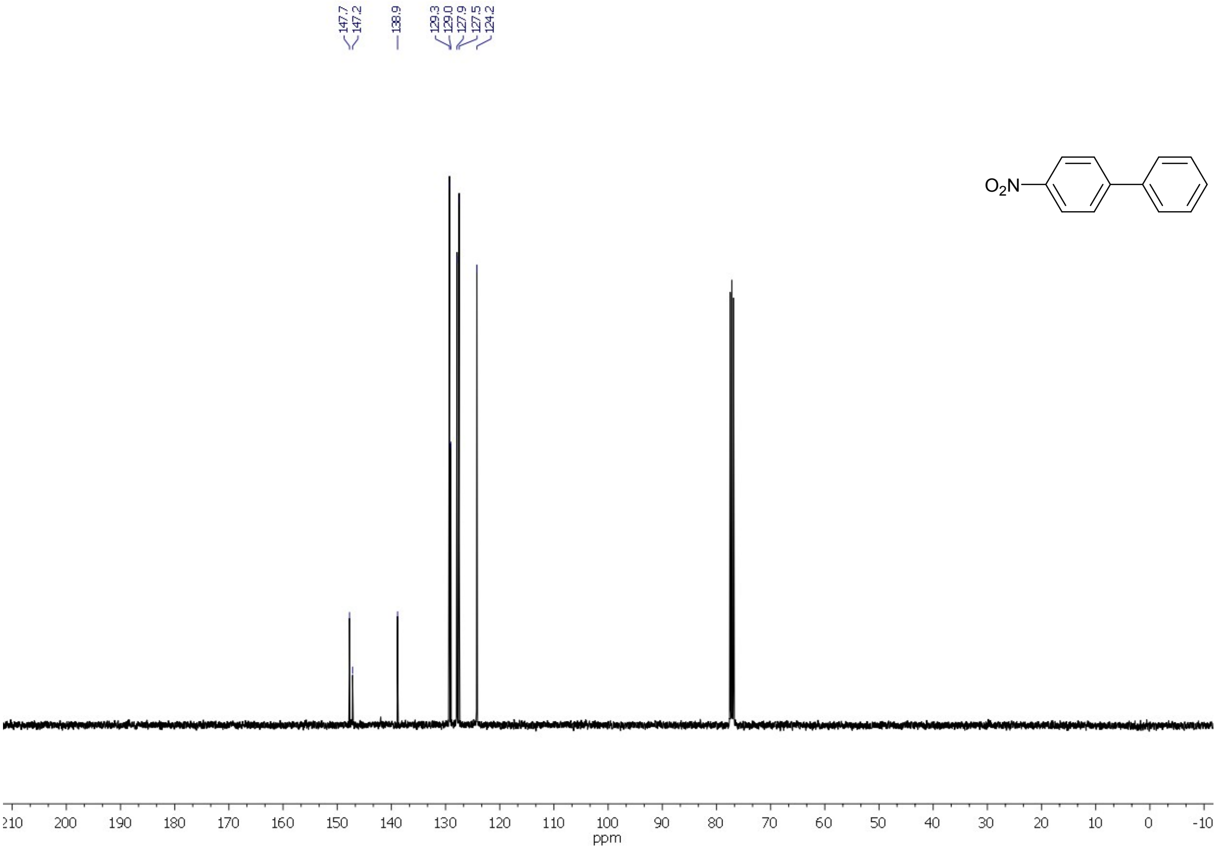

Figure S29: ^{13}C NMR of compound **2j** ($CDCl_3$, 101 MHz).

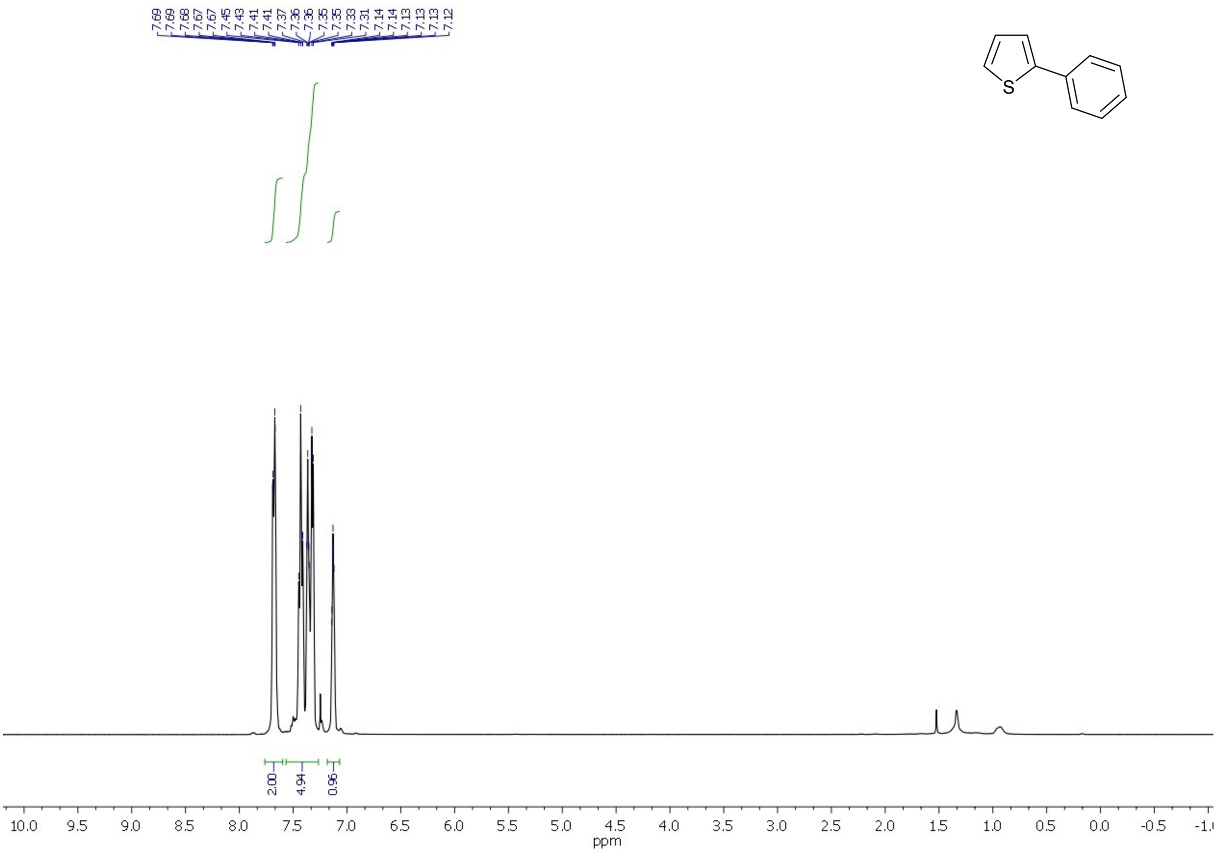

Figure S30: ^{1}H NMR of compound **2k** ($CDCl_3$, 400 MHz).

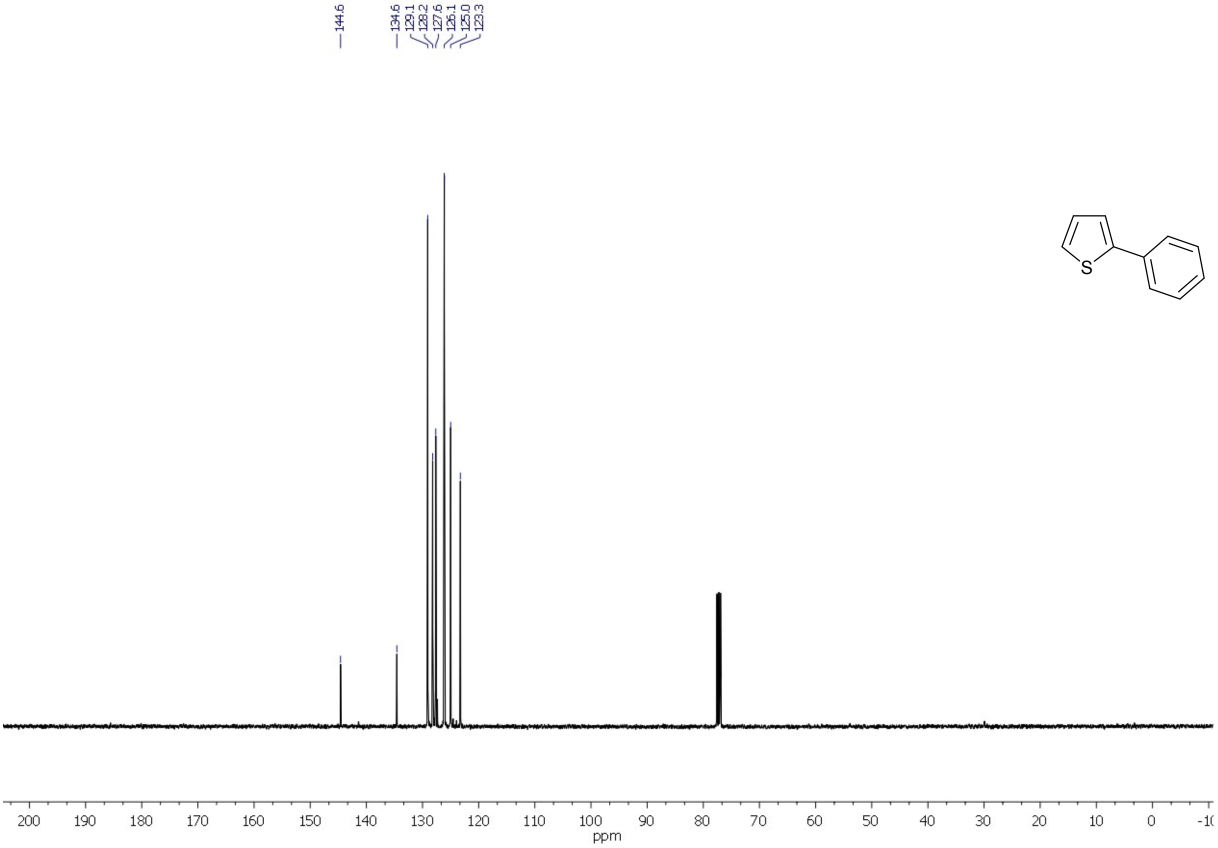

Figure S31: ^{13}C NMR of compound **2k** ($CDCl_3$, 101 MHz).

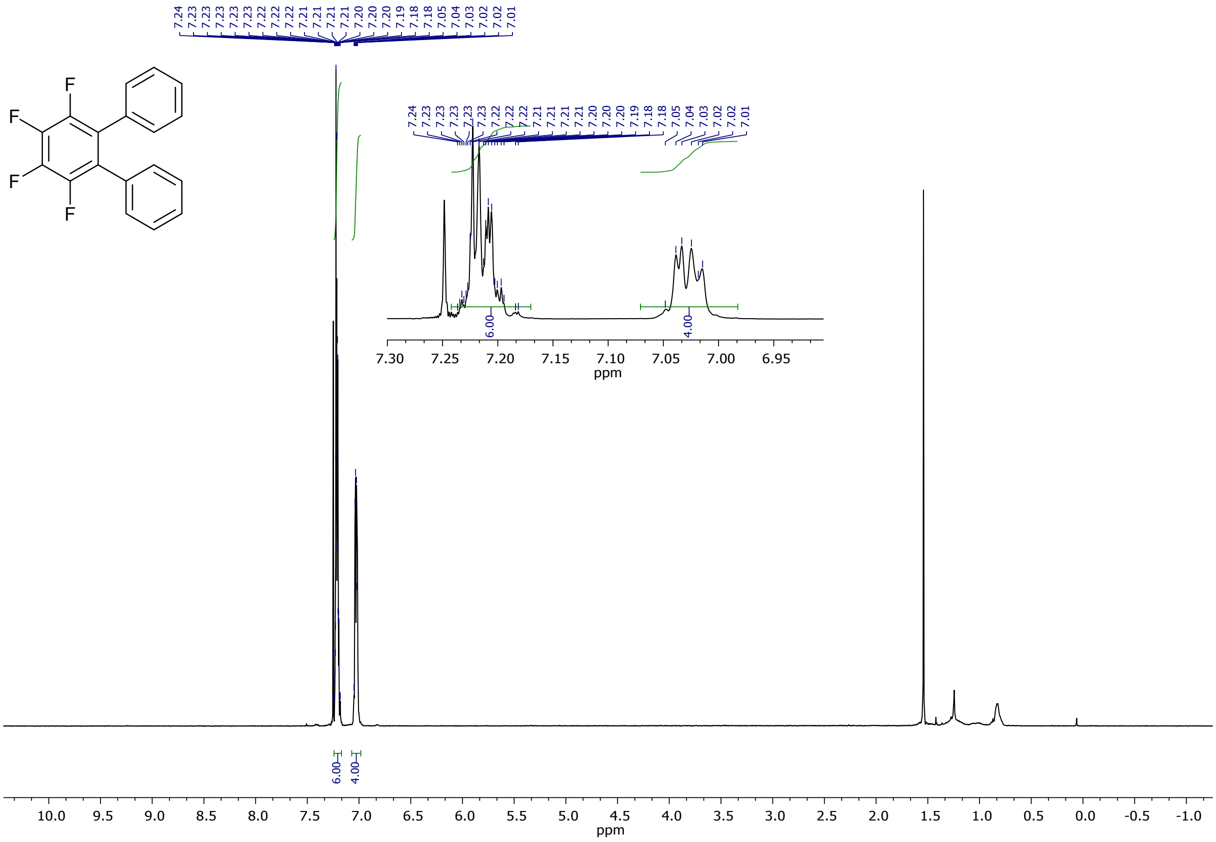

Figure S32: ^{1}H NMR of compound **2l** ($CDCl_3$, 400 MHz).

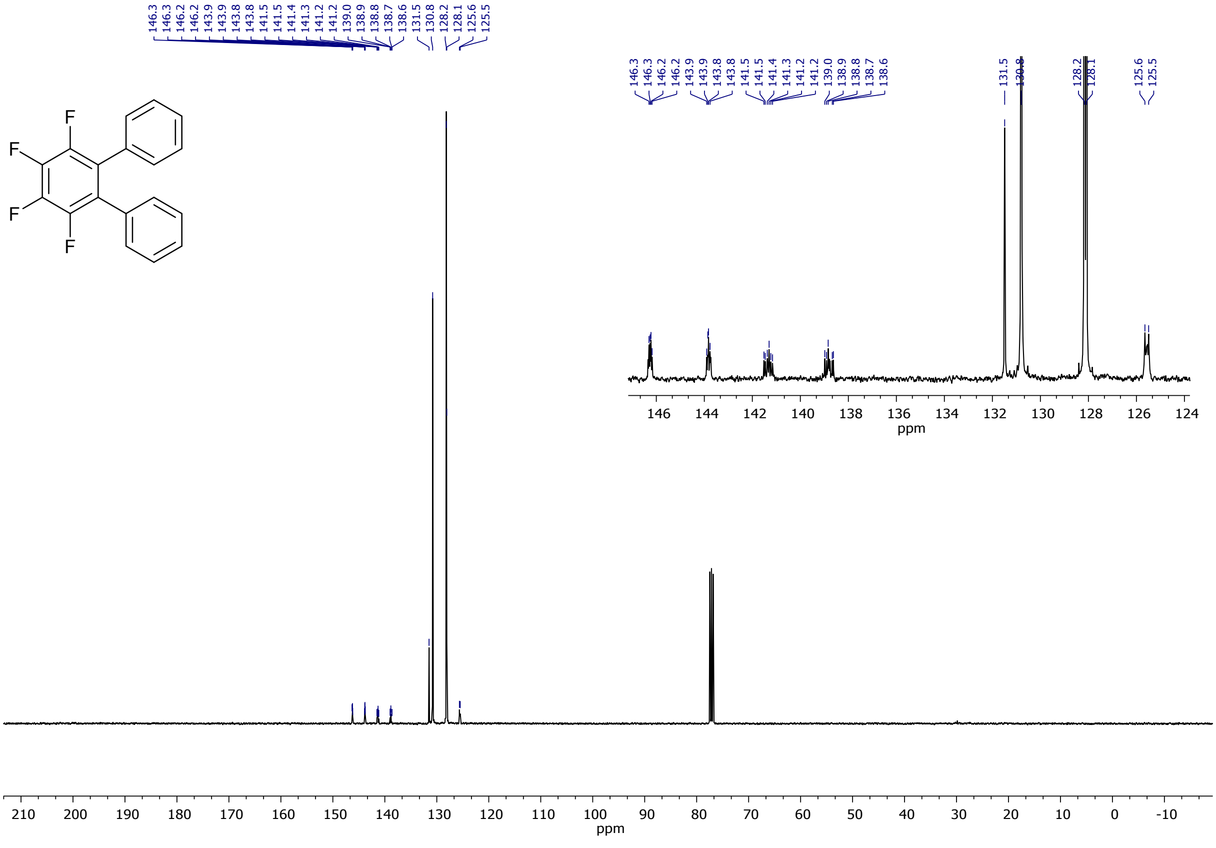

Figure S33: ^{13}C NMR of compound **2l** ($CDCl_3$, 101 MHz).

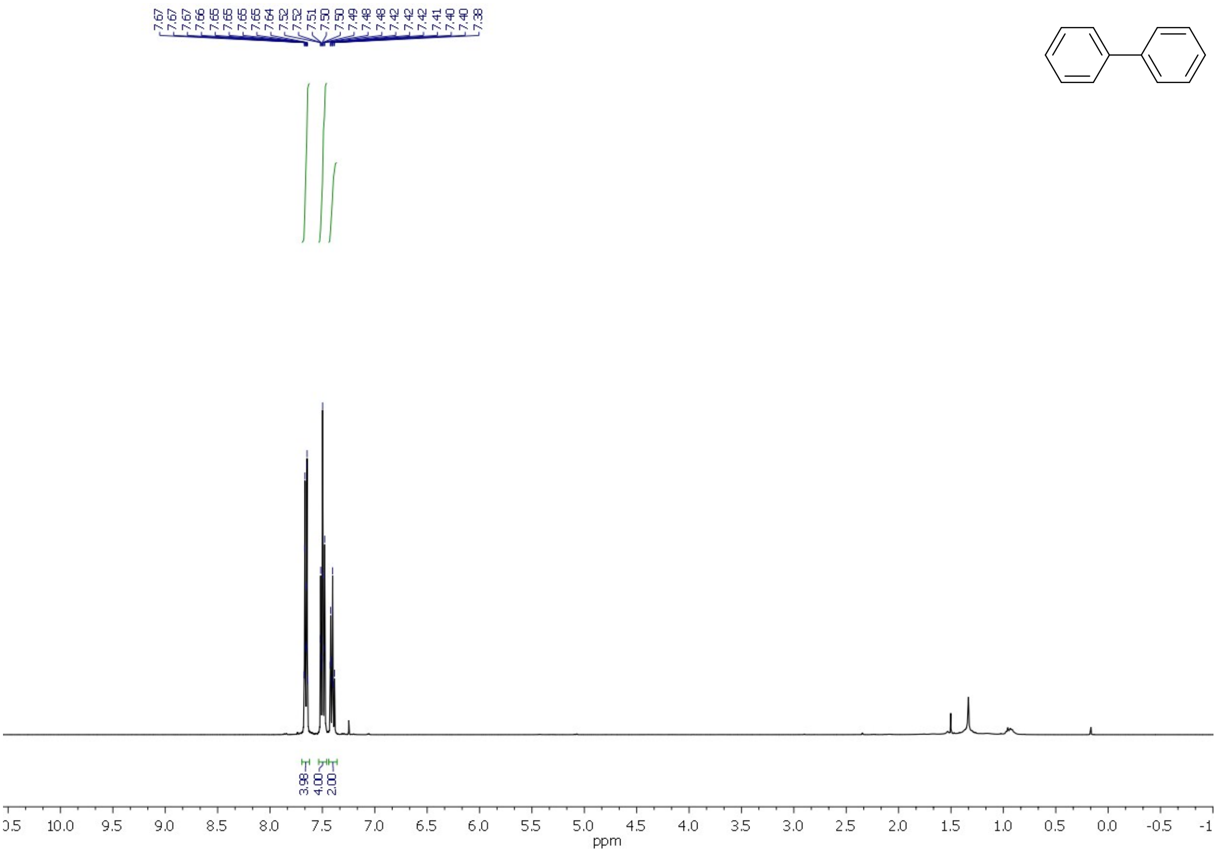

Figure S34: ^{1}H NMR of compound **2m** ($CDCl_3$, 400 MHz).

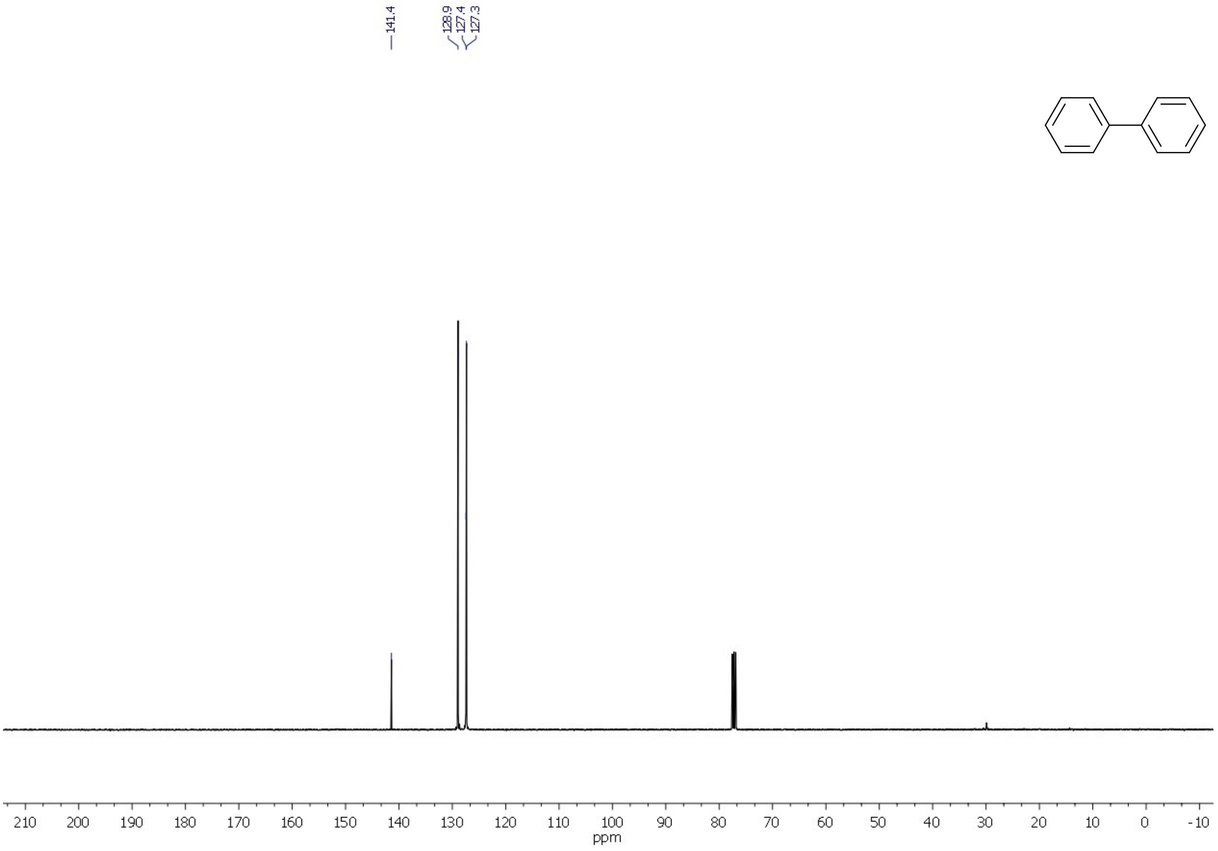

Figure S35: ^{13}C NMR of compound **2m** ($CDCl_3$, 101 MHz).

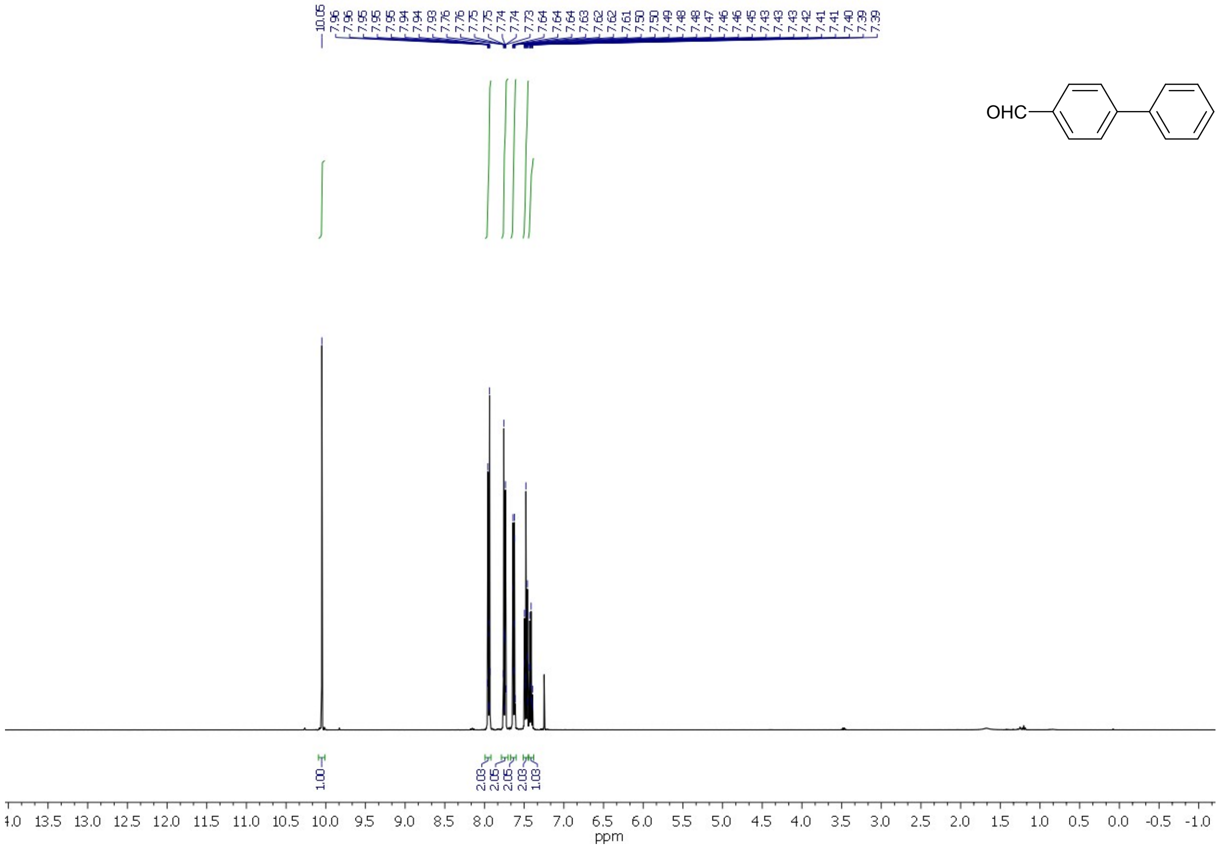

Figure S36: ^{1}H NMR of compound **2n** ($CDCl_3$, 400 MHz).

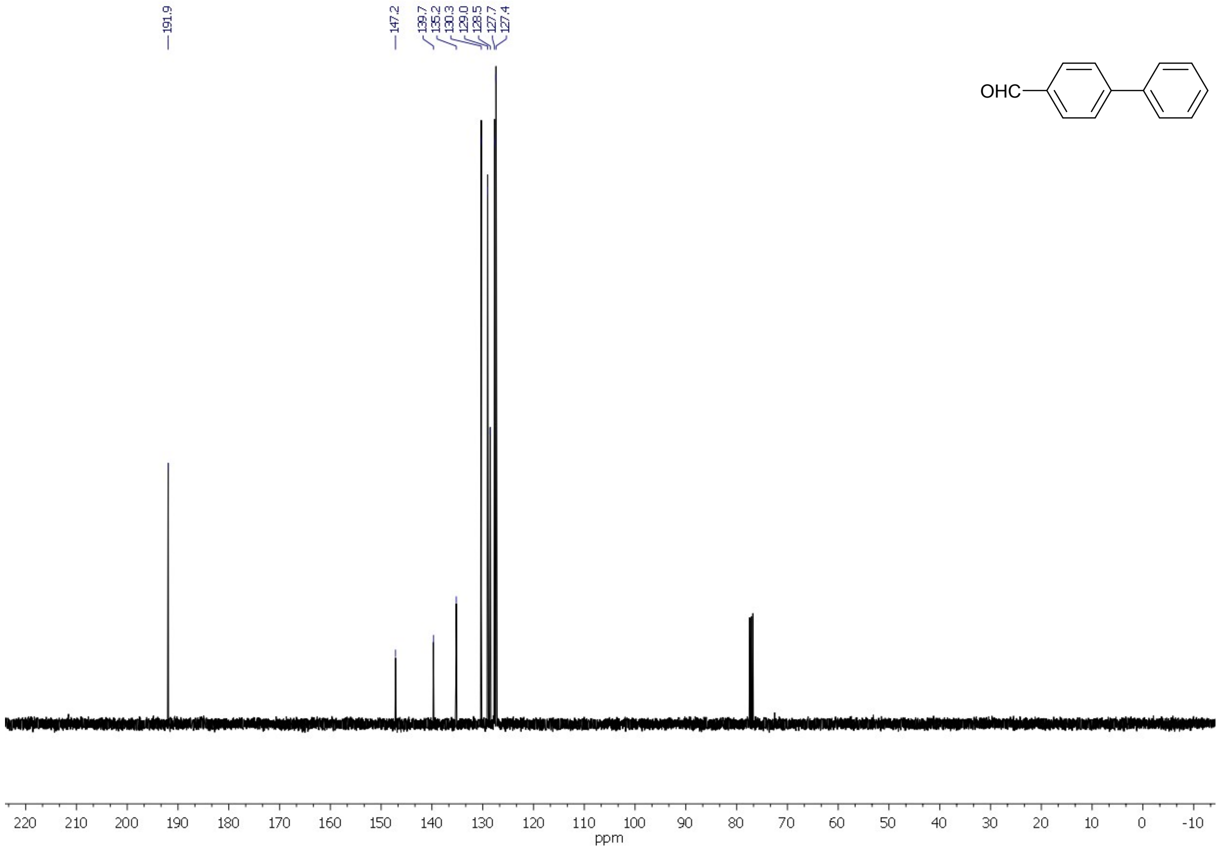

Figure S37: ^{13}C NMR of compound **2n** ($CDCl_3$, 101 MHz).

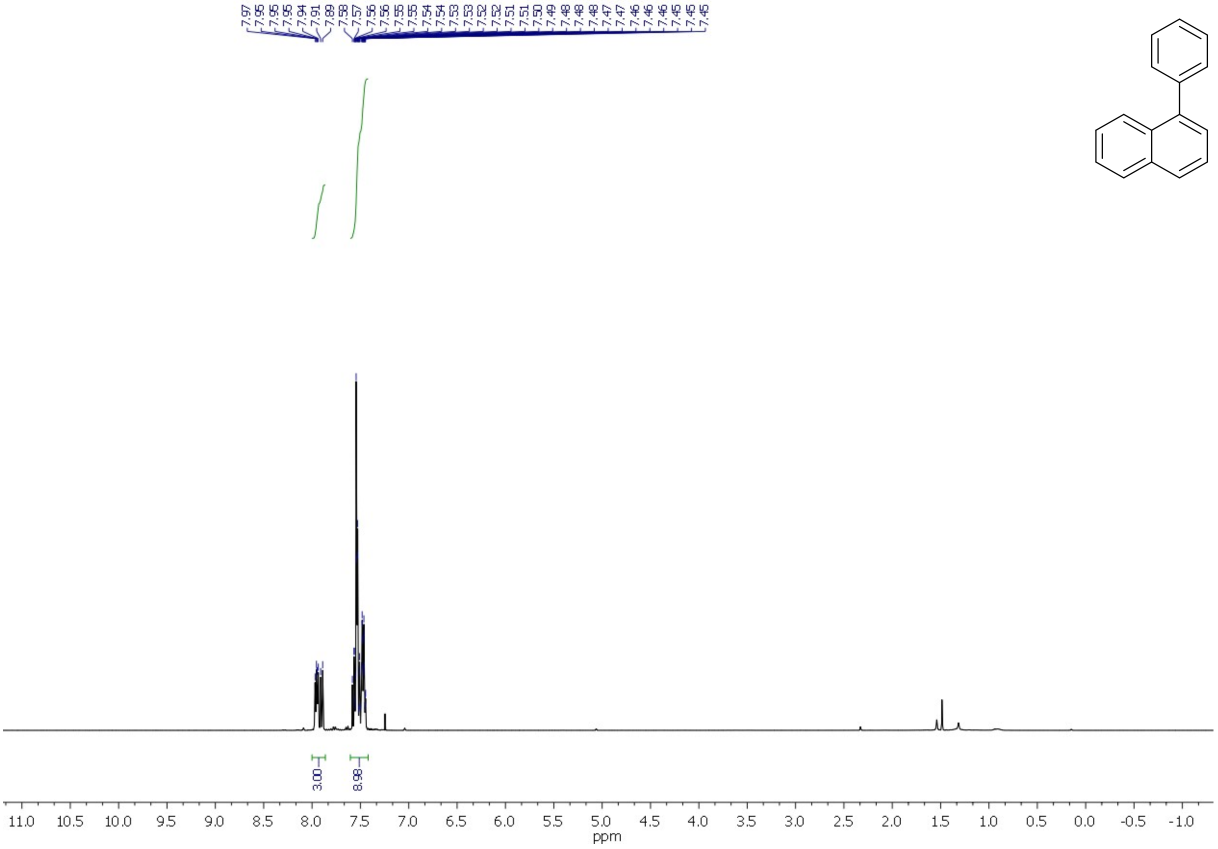

Figure S38: ^{1}H NMR of compound **2o** ($CDCl_3$, 400 MHz).

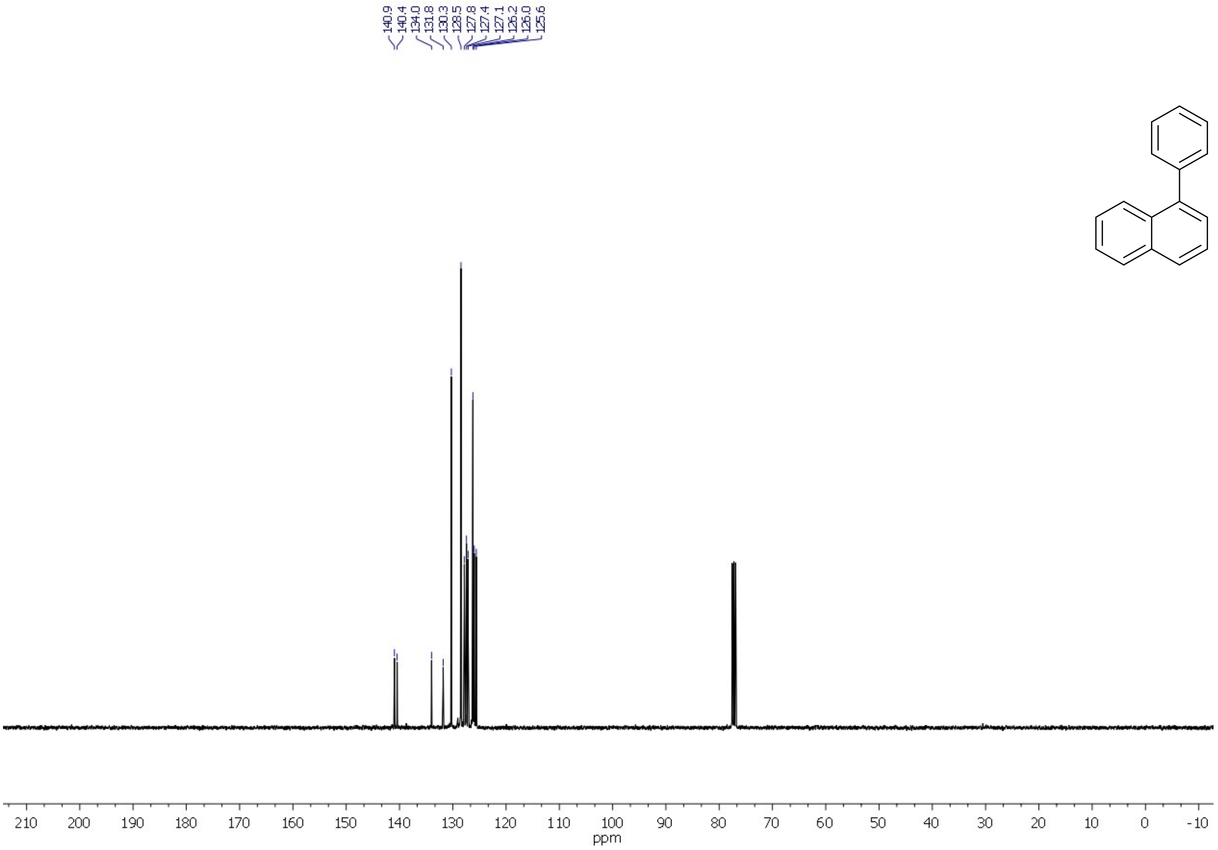

Figure S39: ^{13}C NMR of compound **2o** ($CDCl_3$, 101 MHz).

12. References

1 B. O. Okesola and D. K. Smith, *Chem. Commun.*, 2013, **49**, 11164−11166.

2 F. Mäsing, H. Nüsse, J. Klingauf and A. Studer, *Org. Lett.*, 2018, **20**, 752−755.

3 V. Percec, G. M. Golding, J. Smidrkal and O. Weichold, *J. Org. Chem.,* 2004, **69**, 3447−3452.

4 N. R. Guha, C. B. Reddy, N. Aggarwal, D. Sharma, A. K. Shil, Bandna and P. Das, *Adv. Synth. Catal.,* 2012, **354**, 2911–2915.

5 Y. Jingxun, L. Jun, S. Guangfa, S. Changdong and Z. Yanghui, *Angew. Chem. Int. Ed.,* 2015, **54**, 4079−4082.

6 L. Zhang, Z. Su, F. Jiang, Y. Zhou, W. Xu and M. Hong, *Tetrahedron* 2013, **69**, 9237−9244.

7 B. Gabriele, R. Mancuso, L. Veltri, V. Maltese and G. Salerno, *J. Org. Chem.*, 2012, **77**, 9905−9909.

8 D. M. Lemal, *J. Org. Chem.,* 2016, **81**, 4931−4938.
